# Trends in Temperature-associated Mortality in São Paulo (Brazil) between 2000 and 2018: an Example of Disparities in Adaptation to Cold and Heat

**DOI:** 10.1007/s11524-022-00695-7

**Published:** 2022-11-10

**Authors:** Aina Roca-Barceló, Daniela Fecht, Monica Pirani, Frédéric B. Piel, Adelaide C. Nardocci, Paolo Vineis

**Affiliations:** 1 Department of Epidemiology and Biostatistics, School of Public Health, MRC Centre for Environment and Health, Imperial College London, Norfolk Place, London, W2 1PG UK; 2 Protection Research Unit in Chemical and Radiation Threats and Hazards, Department of Epidemiology and Biostatistics, School of Public Health, National Institute for Health Research Health, Imperial College London, Norfolk Place, London, W2 1PG UK; 3 Department of Epidemiology and Biostatistics, School of Public Health, National Institute for Health Research Health Protection Research Unit in Environmental Exposures and Health, Imperial College London, Norfolk Place, London, W2 1PG UK; 4 Department of Environmental Health, School of Public Health, University of São Paulo, São Paulo, Brazil

**Keywords:** Urban health, Health inequalities, Climate change, Temperature, Mortality

## Abstract

Exposure to non-optimal temperatures remains the single most deathful direct climate change impact to health. The risk varies based on the adaptation capacity of the exposed population which can be driven by climatic and/or non-climatic factors subject to fluctuations over time. We investigated temporal changes in the exposure–response relationship between daily mean temperature and mortality by cause of death, sex, age, and ethnicity in the megacity of São Paulo, Brazil (2000–2018). We fitted a quasi-Poisson regression model with time-varying distributed-lag non-linear model (*tv-*DLNM) to obtain annual estimates. We used two indicators of adaptation: trends in the annual minimum mortality temperature (MMT), i.e., temperature at which the mortality rate is the lowest, and in the cumulative relative risk (cRR) associated with extreme cold and heat. Finally, we evaluated their association with annual mean temperature and annual extreme cold and heat, respectively to assess the role of climatic and non-climatic drivers. In total, we investigated 4,471,000 deaths from non-external causes. We found significant temporal trends for both the MMT and cRR indicators. The former was decoupled from changes in AMT, whereas the latter showed some degree of alignment with extreme heat and cold, suggesting the role of both climatic and non-climatic adaptation drivers. Finally, changes in MMT and cRR varied substantially by sex, age, and ethnicity, exposing disparities in the adaptation capacity of these population groups. Our findings support the need for group-specific interventions and regular monitoring of the health risk to non-optimal temperatures to inform urban public health policies.

**Supplementary Information:**

The online version contains supplementary material available at 10.1007/s11524-022-00695-7.

## Introduction

Approximately 10.34% of the annual deaths globally can be attributed to non-optimal temperatures, which is more than 5 million excess deaths annually [1]. In Brazil, the annual mean temperature (AMT) is projected to rise by about 1.8 °C on average from 1990 to 2100, and the number of days of heatwaves to increase from < 10 to 90 days if emissions are successfully reduced (i.e., low emission scenario, RCP2.6) [2]. This would result in a greater number of people at risk of heat-related mortality. Nonetheless, a reduction in the mortality risk associated with temperature, known as *adaptation*, is possible if populations reduce their exposure levels and/or underlying risk. From a public health perspective, understanding how the temperature-related health burden changes over time and across population groups is important to inform the development and implementation of targeted and equitable prevention plans, such as early warning systems [3].

Adaptation can result from physiologic changes (intrinsic adaptation) occurring as a response to the changing temperature, and/or of non-climate driven factors (extrinsic adaptation) such as economic development, behavioral changes, infrastructure improvements, technological advances, preventive initiatives, or improvements in the health-care services, among others. The overall adaptive capacity of a population is likely to be the combination of all these factors. Adaptation to increased temperatures has been reported in many locations [4–10], mostly in developed countries, with fewer and less conclusive results for cold temperatures [6]. However, the magnitude of the adaptation varies substantially by setting, highlighting the importance of the local context and the need for city-specific assessments [11, 12]. Differences are also observed by population groups such as age and sex, exposing disparities in adaptation capacity [7, 8, 13, 14]. Evidence on other population groups such as ethnic or socioeconomic groups is limited [6, 15, 16].

In the last decades, studies assessing temporal changes in the temperature-mortality association have been on the rise [8, 10, 17–24], yet they suffered from several limitations. Firstly, a large proportion of studies assessed changes over time by dividing the study period in either overlapping [8] or non-overlapping time periods [25], substantially hindering the statistical power to detect differences. Studies on cold have been limited and shown conflicting and inconclusive results [21, 26, 27], but as populations adapt to heat, it is important to assess whether this is in detriment to an increased susceptibility to cold [26, 27]. Furthermore, there exists a geographic disparity in the availability of evidence on trends [28], with most studies set in the USA [18, 19], Asia [20, 21, 29], and Europe [8, 16, 22, 23]. Some studies have explored multiple cities [24], yet they are dominated by cities in Western countries. Finally, most city-level studies depict the effect on the whole population, [4], with only few investigating effects by age and sex [7, 8, 13, 14], and less so for vulnerable populations, such as ethnic groups, education level and/or socioeconomic status [6, 15, 16]. This is mainly due to a lack of individual level information and poor statistical power due to small number of cases. Some studies have attempted to address the latter by using pooled estimates across cities [24], in detriment to capturing the individuality of each city. With more than 11.3 million inhabitants, São Paulo is the largest city in Brazil and suffers from both the effects of cold and heat [30–32]. To date, no study has assessed the local temperature adaptation patterns in São Paulo except for a multi-city study which pooled the estimates for multiple cities in Brazil together [26].

The present study investigates trends in the temperature-mortality association by sex, age, cause of death, ethnicity, and years of education in the megacity of São Paulo, Brazil, between 2000 and 2018. We explored both changes in the cumulative relative risk (cRR), and the minimum mortality temperature (MMT), providing a comprehensive summary of the evolution of the health burden of temperature in the city. In doing so, we provide information that can support policymakers and stakeholders to detect vulnerable groups and potential drivers of change, helping them to manage and reduce the prevalence of temperature-related deaths in the present and in the future.

## Methods

### Study Area and Meteorological Characteristics

**Fig. 1:**
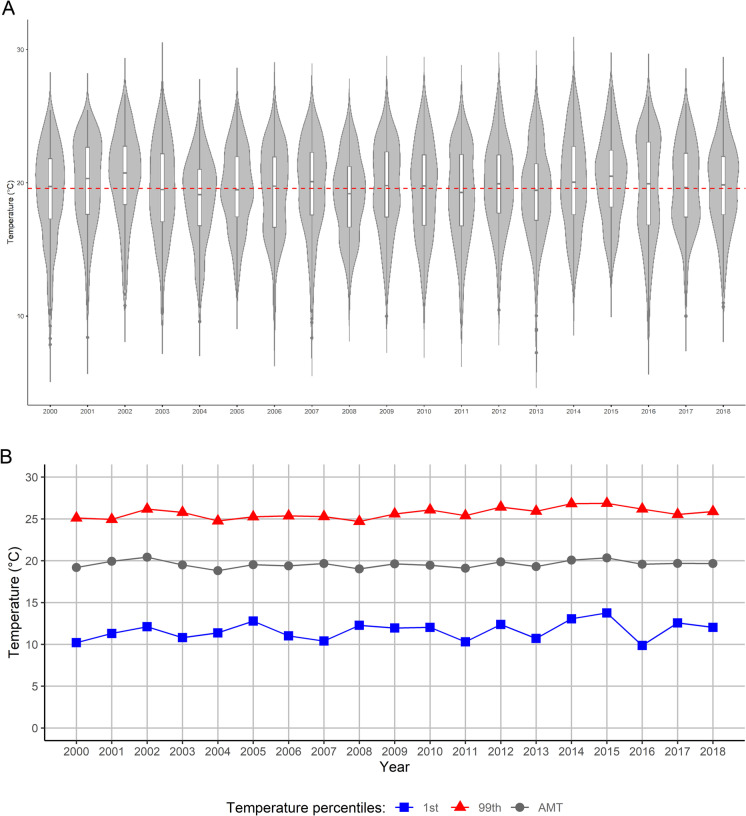
(A) Daily mean temperature distribution by year. In red, the average temperature for the entire study period (2000–2018). (B) Trends in the 99^th^ (red), annual mean temperature AMT (grey) and 1^st^ (blue) percentile of the annual temperature distribution

São Paulo is one of 645 municipalities in the State of São Paulo, South-Eastern Brazil (Fig. 1). It covers 1521.1 km^2^ and has approximately 11.3 million inhabitants (population density: 7398.3 people/km^2^) based on the 2010 census [33]. According to the Köppen-Geiger classification, it has a humid subtropical climate in the North and a temperate oceanic climate in the South [34].

### Data

#### Mortality and Covariate Data

We obtained daily mortality records for São Paulo between 2000 and 2018 from the national Public Health System database (DataSUS) of the Ministry of Health of Brazil. Each record included information on cause and date of death, age, sex, ethnicity, and years of education. We investigated the following causes of death, as defined by the International Classification of Disease, 10th edition (ICD-10): all non-external causes (ICD10: Chapters I-XVIII, Block A00-R99); cardiovascular diseases, CVD (ICD10: Chapter IX, Block I00-I99), and respiratory diseases (ICD10: Chapter X, Block J00-J99). Death certificates in Brazil are compulsory, ensuring population coverage.

Counts were stratified by age (65–79 and ≥ 80 years old), sex (female and males), ethnic group (white and non-whites), and years of education (≤ 3, 4–11, and > 11 years). For age, we focused on the eldest groups because of higher vulnerability [35]. For sex, we used a binary sex classification as a more inclusive classification was not available. Ethnic groups as defined in the 2010 census were grouped in white (census term: *Branco*) and non-white, which included Black (*Preto*) and Mixed ethnicity (*Pardo*). East Asians (*Amarelo*) and Indigenous (*Indígena*) were excluded of this group as they have been shown to have different risk profiles and they had too small counts (*n* = 67,782) to be considered individually. Years of education was only explored for years after 2011, due to a high proportion of missing data in prior years (Supplementary information 1, SM1 and Table S1). Records with missing data were excluded from group-specific analyses after ensuring absence of bias through a chi-square Pearson statistic test (Table S2).

#### Meteorological and Air Pollution Data

Hourly temperature (°C) and relative humidity (RH; %) data were obtained from the Institute of Astronomy Geophysics and Atmospheric Sciences and University of São Paulo (IAG-USP) meteorological station (coordinates: 23,6512°S, 46,6224°W; elevation: 799.2 m, Figure S1). Hourly concentrations of particulate matter with diameter ≤ 10 µm (PM_10,_ µg/m^3^) were obtained from the regional Environment Agency (CETESB). Only stations with ≥ 75% valid data were included (18 out of 36 stations). We estimated the daily mean temperature, RH and PM_10_ as the 24 h concentration average of hourly measurements. For PM_10_, these were then averaged across all valid stations. Refer to the Data Sharing section and SM2 for more information.

### Statistical Analyses

To assess the temporal trends, we estimated the annual temperature-mortality association through a quasi-Poisson regression and time-varying distributed lag non-linear models (*tv*-DLNMs). All analyses were performed in R software (version 3.6.3; R Development Core Team), using functions from the package *dlnm* [36]. *P*-values < 0.05 were considered statistically significant. Interactive visualizations and R scripts are available on the project’s website (https://ainarb.github.io/climate_and_health/) and GitHub (https://github.com/AinaRB), respectively.

#### Temperature Trends

We visually investigated the temporal variability in the temperature distribution (Fig. 1A and S3) and the trends in the annual 1^st^ and 99^th^ percentile of temperature, representing extreme cold and heat, and the AMT (Fig. 1B). We formally tested for trends using linear regression with year as the independent variable.

#### The Distributed Lag Non-linear Model (DLNM) Framework

The effects of temperature on mortality are non-linear, with the highest mortality risks occurring at the extremes of the temperature distribution, and are lagged or delayed, meaning that the impacts expand over several days. DLNMs are a class of models that allow for complex non-linear and lagged dependencies by using a bi-dimensional space of functions, describing the exposure–response association and the lag-response association. These basis functions are combined through a tensor product, resulting in a bi-dimensional crossbasis function describing the exposure-lag-response association. A full description of the DLNM modeling framework, algebraic notation and applications can be found in Gasparrini et al. 2010 [37]. The standard DLNM models assume a time-constant exposure-lag-response association. In this study, however, we are interested in assessing trends. Hence, we used the time-varying DLNM (*tv*-DLNM) [10] extension which includes a linear interaction between time and the crossbasis of temperature to allow the exposure-lag-response association to vary over time in a linear manner.

#### Model Specification

We estimated temperature-mortality associations using a quasi-Poisson regression model incorporating a *tv*-DLNM to capture a non-linear and delayed association varying over time. The final model formulation is a result of fitting 23 competitive analyses with alternative model parametrization and selecting the best model fit based on the quasi-Akaike Information Criterion (qAIC) [38]. We performed our analyses by cause of death (CVD and respiratory diseases) and for all non-external causes of death stratified by sex, age, ethnicity, and combinations of those covariates.

Our model assumed the following general form:

1 $$\mathrm{log}\left[\mathrm{E}\left({\mathrm{Y}}_{\mathrm{t}}\right)\right]=a+{cb}_{{temp}_{t}}+{\sum }_{k=1}^{7}{\beta }_{k}I\left({dow}_{t}=k\right)+\gamma {holidays}_{t}+\delta {PM}_{{10}_{{lag0-2}_{t}}}+ns\left({RH}_{{lag0-2}_{{t}^{^{\prime}}}}3df\right)+ns\left(time,10df\;x\;year\right)+{cb}_{{temp}_{t}}\times\;time;for\;t=1\;...\;T=\mathrm{6,940}$$

where ${Y}_{t}$ denotes the mortality counts at day *t*, *α* is the intercept, and *cb*_*temp*_ is the bi-dimensional crossbasis matrix. The crossbasis function of daily mean temperature was composed of a natural cubic spline (*ns*) with one knot at the 75^th^ percentile of the temperature distribution for the exposure dimension, and a *ns* with an intercept and three knots equally spaced over the log scale of the temperature distribution for the lag dimension. We set the maximum lag effect to expand over 21 days to capture the delayed effects and short-term harvesting [39].

To eliminate potential confounding by time-varying factors, we included the following covariates: a time variable to account for long-term trends and seasonality modeled as a *ns* with 10 df per year [$ns\left(time, 10df x year\right)]$; categorical variables for day-of-the-week [*dow*], and national and statewide holidays [*holidays*], and the ns of the 2-day moving average of the daily mean RH with 3df [$ns\left({RH}_{{lag0-2}_{t}}, 3df\right)]$ and the 2-day moving average of the daily mean PM_10_ value [ ${PM}_{{10}_{{lag0-2}_{t}}}$].

Finally, to allow for time-varying exposure-lag-response associations, we added a linear interaction term between time and the temperature crossbasis variable [${cb}_{{temp}_{t}}\times time$]. It represents the exposure-lag-response relationship at the centring point of the time variable used in the interaction term. Here, we were interested in annual changes in the temperature-lag-mortality association; thus, we used the central day of each year (1^st^ July) to obtain equidistant estimates summarizing each year. Based on the linear assumption of the interaction term, the effect at that point represents the average effect of the year. To formally assess the significance of the interaction, we used a multivariate Wald test on the interaction coefficients, considering the associated (co)variance matrix and assuming a multivariate normal distribution [17]. The null hypothesis is that all coefficients are equal to zero, meaning that there is no change. In addition, we explored the visual representation of the interaction terms.

To evaluate the impact of model choices in our results, we conducted 23 sensitivity analyses modifying the specification of the parameters. See SM3 and Table S4 for details. The model with the lowest qAIC score was selected as the main model and its results are hereafter described in detail. Finally, to evaluate the robustness of our results against alternative model parametrizations, we estimated annual MMT values (Fig. S12) and cRR estimates (Fig. S13-14) resulting from all sensitivity analyses. We found no significant change, supporting the robustness of our model.

#### Indicators of Adaptation: Time-varying MMT and cRR

To assess adaptation, we explored fluctuations in the MMTs and cRRs. The cRR represents the overall effect associated with a given day’s temperature over a maximum lag period, in our case, of 21 days. The association follows a V or U-shape. The temperature at which the mortality risk is the lowest is referred to as the MMT and it is often considered a good indicator for long-term adaptation to local climate [23, 40, 41]. The slope of the cRR curve defines the increment of risk per unit of temperature. The steeper the slope, the larger the increase in risk per unit of temperature. Hence, a decrease in the slope (or flattening of the curve) over time indicates a reduction in the risk increment per unit of temperature change. Accordingly, we used changes in MMT and in the cRR associated to extreme heat (99^th^ percentile), moderate heat (90^th^ percentile), moderate cold (10^th^ percentile) and extreme cold (1^st^ percentile) as indicators of adaptation. Refer to SM4 for a detailed description and visual representation of the indicators.

To estimate both indicators, we reduced the lagged association cumulating the risk over the 21 days of maximum lag. For the MMTs, we estimated the annual value for each group and the associated 95% empirical confidence intervals (eCI) and standard error using a parametric bootstrap estimator [42]. We considered only temperatures above the 1^st^ and below the 99^th^ percentile of the group-specific temperature distribution, known as *percentile constricted MMT*, to avoid unstable cRR curve tails affecting the computation of the MMT [42]. Hereafter, when we talk about MMT, we are referring to the percentile constricted MMT. We used these year- and population-specific MMTs to re-center the corresponding cRR curves.

For all indicators, we evaluated the temporal trends using a weighted least square linear regression with year as the independent variable. This method corrects for non-constant variance by weighting each observation by penalizing observations with large variance. We evaluated the goodness of fit (*R*^2^), the Snedecor *F* value and inspected the plot of residuals vs the fitted values to assess the plausibility of the linearity assumption. Finally, we assessed the association between the annual MMT and AMT, cRR for extreme heat and annual 99^th^ percentile of temperature, and cRR for extreme cold and the 1^st^ percentile of temperature, using the same method. For each pair, we provide the regression coefficients, Pearson Correlation coefficient and *p*-value.

### Ethics and Governance

The study was approved by the ethics committee of the University of São Paulo (CAAE: 31,664,820.0.0000.5421) and conducted in accordance with Brazilian Law 13,709/2019 on the protection of personal data.

## Results

### Temporal Trends in Meteorological and Mortality Data

Table S3 provides descriptive statistics for mortality counts and environmental variables by population group for the overall period (2000–2018) and for first (2000–2004) and last 5 years (2014–2018). Between 2000 and 2018, the average daily mean temperature and RH were 19.6 °C (min: 7.3 °C, max: 28.0 °C) and 80.0% (min: 34.3%, max: 97.3%), respectively. Temperatures showed a clear seasonality (*p*-value < 0.05), with the four hottest months being December–March, and the coldest May–August (Fig. S2). During the study period, there were four El Niño (2002–2003, 2005–2006, 2009–2010, and 2015–2016) and three La Niña strong events (2007–2008, 2010–2011, and 2018–2019). Yet, we found no strong pattern in our temperature data (Fig. 1A and Fig. S3). The AMT fluctuated by ± 2 °C with respect to the study period mean, yet with no clear trend. The frequency of extreme temperatures fluctuated, yet with no clear trend neither (Fig. 1B). Overall, 4,471,000 deaths were recorded for all non-external causes, of which 1,492,164 were from CVD and 614,959 from respiratory diseases (33.4% and 13.8%, respectively). Of all non-external causes of death, 53.6% occurred among men, 70.0% among white population, and 28.0% among the ≥ 80 years old. Mortality counts increased across all groups (Fig. S4a–d and Fig. S4) as expected considering the increase in population during the study period.

### Indicators of Adaptation

**Fig. 2:**
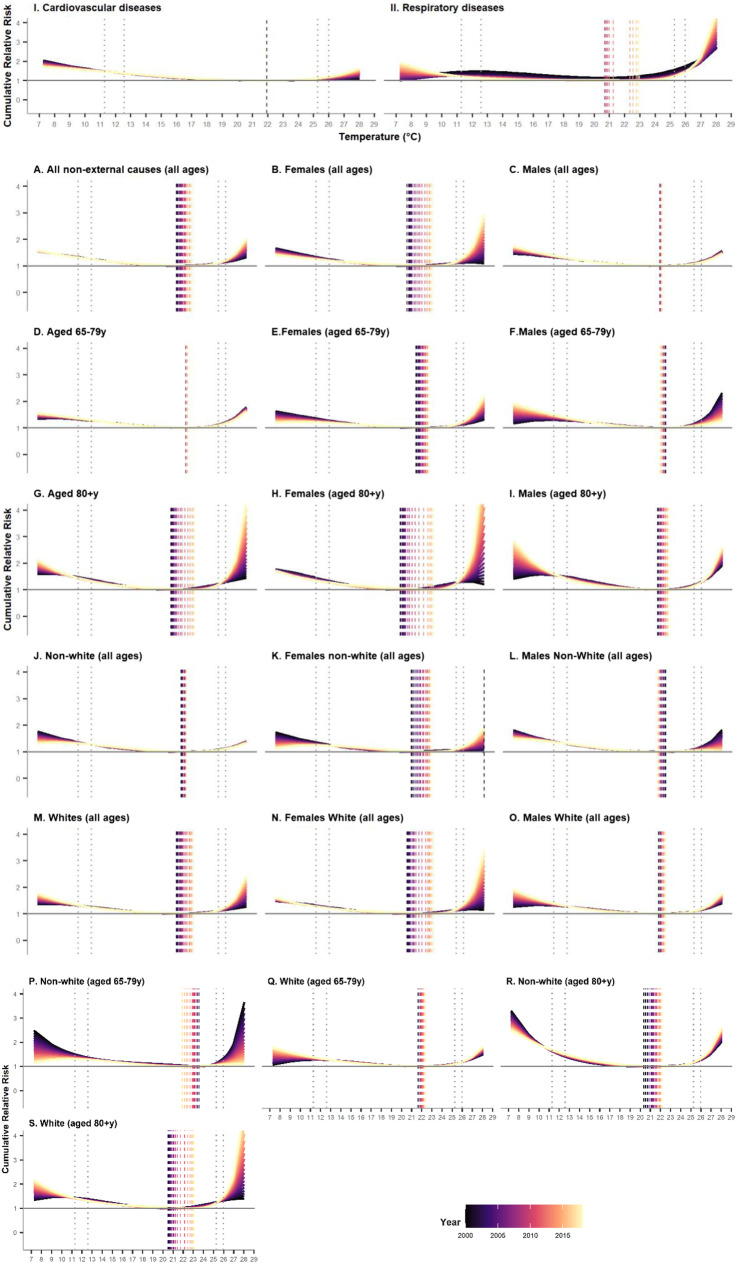
Annual cumulative relative risk (cRR, solid line) for the temperature-mortality association for (I) cardiovascular diseases (CVD) mortality and (II) respiratory diseases mortality, and for (A–S) all non-external causes of deaths by age, sex, ethnic groups, and combination of those. The annual MMT shown as a vertical dashed line. The 1^st^, 10^th^, 90^th^ and 99^th^ percentiles of the temperature distribution are shown as dotted vertical lines

Figure 2 displays the annual cRR curves with their MMTs (dashed vertical lines) by population group (models with interaction). A graphic representation of the interaction can be found in Fig. S7. The overall cumulative exposure–response curves for the entire study period (model with no interaction) can be found in Fig. S6. Analyses by education level showed small and insignificant changes, with large and overlapping 95% CI (Fig. S8) and were not explored further. Table 1 contains the MMT, and the cRR (95%CI) estimates at the 1^st^ and 99^th^ percentiles predicted for the first (2000) and last (2018) years of the study period.

**Table 1:** Temporal changes in the MMT and cRR associated to extreme and moderate heat and cold by population group for the first (2000) and last year (2018) of the study period. The 95% CI are provided for all estimates. Extreme heat and cold defined as the 99^th^ and 1^st^ percentile of the annual temperature distribution estimated using the annual specific MMT as the reference value, respectively. *P*-value of the Wald test for interaction shown in the last column, marked with (*) those statistically significant (*p*-value < 0.05)

Population group		Year	Extreme cold ^a^		Moderate cold ^b^		Moderate heat ^c^		Extreme heat ^d^		Wald test	
			cRR (95% CI)		cRR (95% CI)		cRR (95% CI)		cRR (95% CI)			
Overall		2000	1.33	(1.27;1.39)	1.14	(1.11;1.17)	1.04	(1.01;1.07)	1.14	(1.06;1.23)	0.00	*
		2018	1.31	(1.25;1.38)	1.14	(1.11;1.18)	1.01	(1.00;1.03)	1.22	(1.16;1.28)		
Gender	Females	2000	1.34	(1.26;1.43)	1.12	(1.08;1.16)	1.06	(1.02;1.11)	1.12	(1.01;1.24)	0.01	*
		2018	1.25	(1.16;1.34)	1.13	(1.08;1.18)	1.02	(1.00;1.03)	1.33	(1.24;1.42)		
	Males	2000	1.33	(1.25;1.40)	1.16	(1.12;1.20)	1.03	(1.00;1.06)	1.17	(1.06;1.29)	0.00	*
		2018	1.38	(1.30;1.47)	1.16	(1.11;1.20)	1.02	(1.00;1.04)	1.13	(1.07;1.20)		
Age group	65–75 years old	2000	1.32	(1.22;1.42)	1.17	(1.12;1.23)	1.03	(0.99;1.06)	1.21	(1.06;1.38)	0.51	
		2018	1.35	(1.25;1.47)	1.17	(1.11;1.23)	1.02	(0.99;1.04)	1.16	(1.08;1.25)		
	≥ 80 years old	2000	1.48	(1.36;1.61)	1.20	(1.14;1.27)	1.12	(1.05;1.18)	1.29	(1.12;1.49)	0.00	*
		2018	1.45	(1.33;1.57)	1.18	(1.12;1.24)	1.02	(1.00;1.03)	1.46	(1.34;1.58)		
Ethnic group	White	2000	1.31	(1.24;1.37)	1.15	(1.11;1.19)	1.05	(1.01;1.08)	1.14	(1.05;1.24)	0.00	*
		2018	1.33	(1.26;1.41)	1.13	(1.09;1.17)	1.01	(1.00;1.02)	1.27	(1.20;1.34)		
	Non-white	2000	1.36	(1.23;1.50)	1.14	(1.07;1.21)	1.02	(0.97;1.08)	1.13	(0.95;1.33)	0.04	*
		2018	1.31	(1.19;1.44)	1.18	(1.11;1.25)	1.02	(0.99;1.05)	1.11	(1.02;1.21)		
Gender and age group	Females aged 65–79 y	2000	1.35	(1.22;1.51)	1.15	(1.07;1.23)	1.03	(0.97;1.10)	1.13	(0.94;1.35)	0.45	
		2018	1.21	(1.07;1.36)	1.13	(1.05;1.21)	1.02	(0.99;1.05)	1.25	(1.12;1.39)		
	Females aged ≥ 80 y	2000	1.45	(1.30;1.62)	1.15	(1.09;1.22)	1.18	(1.08;1.28)	1.30	(1.09;1.56)	0.02	*
		2018	1.35	(1.21;1.50)	1.17	(1.09;1.25)	1.02	(1.01;1.04)	1.58	(1.42;1.75)		
	Males aged 65–79 y	2000	1.29	(1.16;1.43)	1.20	(1.12;1.28)	1.02	(0.99;1.06)	1.28	(1.08;1.53)	0.50	
		2018	1.49	(1.34;1.65)	1.20	(1.13;1.28)	1.02	(0.98;1.06)	1.10	(1.00;1.21)		
	Males aged ≥ 80 y	2000	1.55	(1.36;1.76)	1.31	(1.21;1.43)	1.06	(0.98;1.13)	1.30	(1.03;1.63)	0.10	
		2018	1.60	(1.41;1.81)	1.19	(1.1;1.29)	1.02	(0.99;1.04)	1.29	(1.14;1.46)		
Gender and Ethnic group	Females non-white	2000	1.35	(0.40;4.62)	1.13	(0.32;3.98)	1.05	(0.29;3.87)	1.07	(0.38;2.99)	0.47	
		2018	1.25	(1.08;1.44)	1.18	(1.08;1.29)	1.01	(0.99;1.04)	1.20	(1.04;1.38)		
	Females white	2000	1.32	(1.22;1.41)	1.13	(1.08;1.18)	1.08	(1.02;1.13)	1.15	(1.02;1.30)	0.06	
		2018	1.27	(1.18;1.38)	1.12	(1.07;1.18)	1.02	(1.00;1.03)	1.37	(1.27;1.48)		
	Males non-white	2000	1.39	(1.21;1.60)	1.16	(1.06;1.27)	1.02	(0.97;1.06)	1.19	(0.94;1.50)	0.18	
		2018	1.37	(1.21;1.54)	1.18	(1.10;1.27)	1.02	(0.98;1.08)	1.05	(0.94;1.17)		
	Males white	2000	1.31	(1.22;1.39)	1.18	(1.13;1.23)	1.03	(0.99;1.06)	1.14	(1.02;1.28)	0.00	**
		2018	1.39	(1.29;1.49)	1.14	(1.09;1.19)	1.01	(1.00;1.03)	1.18	(1.10;1.26)		
Ethnic and age group	Non-white aged 65–79y	2000	1.51	(1.21;1.88)	1.22	(1.06;1.40)	1.00	(0.99;1.01)	1.33	(0.94;1.89)	0.75	
		2018	1.30	(1.11;1.54)	1.20	(1.09;1.32)	1.02	(0.96;1.09)	1.08	(0.92;1.26)		
	Non-white aged ≥ 80y	2000	1.62	(1.26;2.09)	1.14	(0.98;1.33)	1.05	(0.87;1.26)	1.26	(0.82;1.94)	0.39	
		2018	1.68	(1.37;2.07)	1.26	(1.11;1.43)	1.03	(0.96;1.10)	1.33	(1.08;1.65)		
	White aged 65–79 y	2000	1.27	(1.17;1.38)	1.19	(1.13;1.26)	1.04	(0.99;1.08)	1.18	(1.03;1.36)	0.28	
		2018	1.38	(1.26;1.52)	1.16	(1.09;1.22)	1.02	(0.99;1.04)	1.19	(1.09;1.30)		
	White aged ≥ 80 y	2000	1.44	(1.32;1.58)	1.22	(1.15;1.29)	1.13	(1.06;1.21)	1.31	(1.12;1.54)	0.00	*
		2018	1.43	(1.30;1.57)	1.17	(1.10;1.24)	1.02	(1.00;1.03)	1.50	(1.37;1.65)		

Abbreviations: *cRR*, cumulative relative risk; *95%CI*, 95% confidence intervals

^a^ Extreme heat: cRR at the 99^th^ percentile vs. MMT

^b^ Moderate heat: cRR at the 90^th^ percentile vs. MMT

^c^ Moderate cold: cRR at the 10^th^ percentile vs. MMT

^d^ Extreme cold: cRR at the 1^st^ percentile vs. MMT

**Fig. 3:**
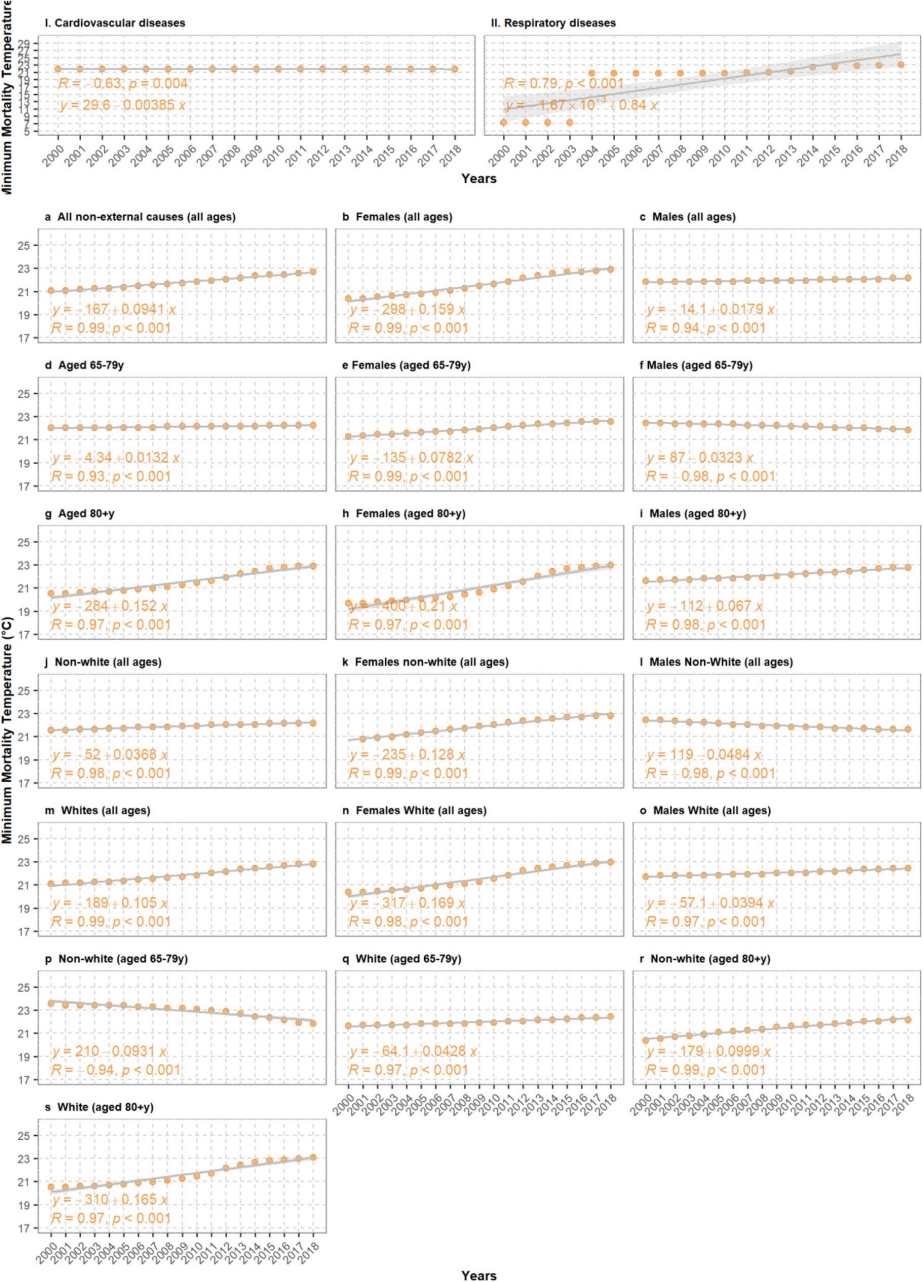
Annual MMT estimates for (I) cardiovascular diseases (CVD) mortality and (II) respiratory diseases mortality, and for (a–s) all non-external causes of deaths by age, sex, ethnic groups, and combination of those. In orange, the fitted linear regression line, regression coefficients, Pearson correlation estimate and *p*-values

Trends in MMT can be found in Fig. 3 with the corresponding WLS regression equation, correlation coefficient and *p*-value, by population group. For all groups, we observed a statistically significant positive linear trend in the MMT estimates, except males aged 65–79 years old (WLS regression coefficient: − 0.03), non-white males (− 0.05), and non-white population aged 65–79 years old (− 0.9) groups, which showed a decreasing trend. The largest increase was observed among females aged ≥ 80 years old (+ 0.21), and white females (+ 0.17).

**Fig. 4:**
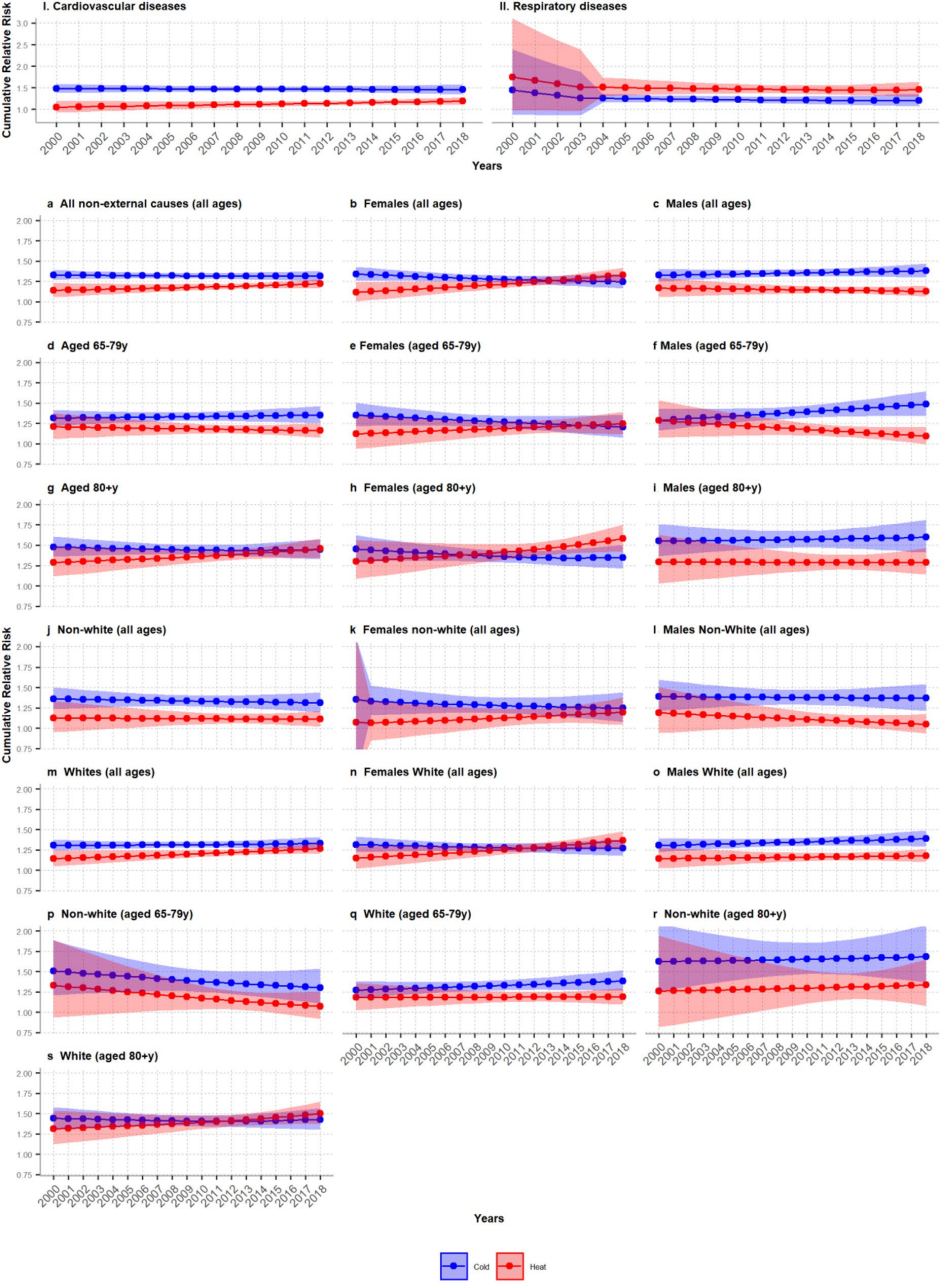
Annual cumulative relative risk (cRR) associated to extreme heat (red) and extreme cold (blue) for (I) cardiovascular diseases (CVD) mortality and (II) respiratory diseases mortality, and for (a–s) all non-external causes of deaths by age, sex, ethnic groups, and combination of those. The 95% CI are shown shaded in the same colour. Extreme heat and cold defined as the 99^th^ and 1^st^ percentile of the annual temperature distribution estimated using the annual specific MMT as the reference value, respectively

Trends in the cRR and 95% CI for extreme cold (1^st^ percentile) and heat (99^th^ percentile) are displayed in Fig. 4. Overall, we observed a statistically significant increase over time in cRR related to heat for all non-external and CVD-related deaths. The cRR associated to cold decreased for CVD deaths. For respiratory diseases deaths, the cRR profile changed remarkably, showing a larger risk to extreme cold, yet a reduced risk to mild heat and cold. For non-external causes of death by population group, the trend was more heterogeneous (Table 1; Fig. 2). All female sub-groups experienced an increase in the cRR for heat and a slight decrease for cold. The reverse was observed for male sub-groups, except for non-white males. The mortality risk to heat and cold increased among those aged ≥ 80 years old, whereas for those aged 65–79 years old, it only increased slightly for extreme cold. Finally, the non-white population showed a decreasing death risk associated with cold, which was not found among the white population.

A formal assessment of the statistical significance of the temporal changes is provided by the Wald test in Table 1. Overall, the time interaction term was statistically significant for all groups, except for people aged 65–79 years old and non-white population, as well as for most of the subgroups. This was most likely due to the larger instability of the estimates due to smaller counts, which also reflects on larger 95% CI.

Finally, we investigated the association of annual MMT, and extreme heat and cold cRRs with AMT, and annual extreme heat and cold, respectively (Figs. S9–S11). For the MMT-AMT pair, we found no statistically significant association (Fig. S9). For the cRR indicator, if adaptation is present, we would expect a decoupling of the association with extreme temperatures, indicating lower risk for higher temperatures. Females and the eldest showed a positive association with extreme heat and a negative association for extreme cold. This suggests that as temperatures are becoming more extreme, so did the risk of death, and so, suggesting lack or poor adaptation, yet the change was small. For the other groups, the association was weak, suggesting a slight decoupling between the extreme temperatures experienced, suggesting some degree of adaptation.

## Discussion

This study investigated trends in the temperature-mortality association by population groups in São Paulo, Brazil, between 2000 and 2018. Three main findings arose from this research. Firstly, that the temperature-mortality relationship is a dynamic process that varies over time. Secondly, that there exists disparities in the presence and magnitude of adaptation across population groups. Finally, that the changes observed seemed to be only partially coupled to changes in different temperature indicators, suggesting the presence of non-climatic adaptation drivers. Below, we describe in detail each of them in the context of the existing literature.

For some groups, we observed a mild negative trend in the mortality burden associated to heat which would be in line with the increase in the annual MMT, suggesting adaptation to higher temperatures. Nonetheless, we observed a slight decrease in the cold mortality burden for some groups despite the annual increase of MMT. The trends for heat and cold are in line with those reported by Vicedo-Cabrera et al.’s study, which included 18 Brazilian cities [26]. They reported a decrease in the RR for heat from 1.095 (1.038; 1.155) to 1.064 (1.015; 1.117), the RR of cold decreased from 1.26 (1.196; 1.347) to 1.222 (1.151; 1.298), between 1997 and 2011. However, the findings were not statistically significant (Wald test: 0.471). When comparing to other countries, our results for heat are in line with the evidence[10]. However, findings for cold are limited and contradicting. Zhao and colleagues [1] reported that a global slight increase in the excess death for cold was found between 2000 and 2011, globally. The decoupling of MMT and risk of death for cold observed in our study, seems to suggest the presence of factors other than temperature changes. This is in line with findings reported in Vicedo-Cabrera et al.’s study [26]. Nonetheless, differences in methods, model parametrization and period under study hinder direct comparison of studies.

We also observed a 1.7 °C increase in the MMT for all non-external causes of death of which is in line with findings from previous studies in France [40, 43], Spain [44], Sweden [41], Austria [23], and the Netherlands [22]. Nonetheless, this is in contrast with the findings from Vicedo-Cabrera et al.’s study [26], where the MMT reduced by − 0.4 °C from 1997 to 2011. Differences in the local context, the period under study and modeling choices may explain the conflicting results. The MMT has been shown on several occasions to respond to changes in the mean annual temperature [45]. However, this is not the case for São Paulo, where we did not see any significant change in AMT nor a statistically significant association between the MMT and the AMT. This could indicate the role of non-climatic factors, as suggested in Vicedo-Cabrera et al.’s study where a similar decoupling was observed [26]. The trends differed by population group with a general of increase of > 1 °C for most of the groups, except for males aged 65–79 years old, non-white males, and non-white population aged 65–79 years old. Different penetration rates of these non-climatic factors across population groups may explain the heterogeneity in cRR trends we observed. Such factors may include: improvements in the life expectancy at birth (70.1 years in 2000; 75.7 years in 2018) [33]; increase in health care coverage (from 7.8 to 58.5% between 2000 and 2016 [46]), adoption of healthier lifestyles, e.g., decreased smoking prevalence (from 35.3 in 1989 to 11.3% prevalence among adults ≥ 20 years old in 2017 [47]), among others. Socioeconomic status is also a known effect modifier of the temperature-mortality association [48, 49]. In 2014, Brazil experience an economic recession that caused a doubling in the unemployment rates in São Paulo which most likely modified the exposure pathways to temperature [50]. Our analyses by educational level aimed at exploring the impact of job market and socioeconomic status, but the poor quality of the data hindered its use. Finally, we did not observe a significant change in the AMT nor in extreme temperatures. Nonetheless, we cannot rule out the role of climate-driven physiological and behavioral adaptation, e.g., uptake of air conditioning [4, 51] or raised awareness of weather-related health impacts.

Overall, our findings suggest the need for local, up-to-date and group-specific evidence to inform public health measures. One such measure is early warning systems. These are short-term responsive measures aimed at minimizing the health impacts of extreme temperature activated upon reaching temperatures of risk for health. According to the WHO guidelines [3], the gold standard to define the temperature thresholds of activation is to consider setting-specific associated health outcome. Currently, São Paulo has a cold spell warning system for the homeless population only. It uses pre-defined temperature threshold based on daily minimum temperature (moderate risk: 13 °C; high risk: 10 °C) [52]. Based on our finding, at a daily mean temperature of 10 °C, the general population experienced a 40% increase in mortality. These estimates are not specific for homeless population nor do they explore minimum temperatures and so, are likely underestimating the impact of cold extreme events. Even so, they suggest that São Paulo could substantially benefit from the development and implementation of a local early warning system which considers population groups, and temporal variations in the risk.

Our study has important strengths. Firstly, we provide evidence for multiple population groups at risk including by ethnicity. Secondly, we used *tv-*DLNMs, an advanced modeling technique able to capture complex non-linear and delayed dependencies between temperature and mortality while allowing for temporal variation. Finally, we evaluated adaptation using multiple indicators, providing a more comprehensive overview. Some limitations include the lack of information on underlying mechanisms of adaptation, e.g., housing conditions or air conditioning data. Secondly, we cannot rule out the effect of changes in air pollution levels as only data on PM_10_ was available. Similarly, changes in the influenza epidemics, sunlight exposure or atmospheric pressure could have also confounded our results, for which data were not recorded. Finally, similar to previous studies, we used data from one meteorological station which could be hiding spatial patterns in the distribution of the risk.

## Conclusions

In conclusion, our study provides evidence of disparities in the adaptation capacity across population groups and temperature ranges (cold vs heat). We did not find a clear association between our indicators of adaptation and different temperature indicators over the study period. We argue that non-climatic factors with different penetration rates in the population are likely to have influenced such patterns. Our findings support the need for regular monitoring of the temperature-mortality association by population groups to suitably inform public health interventions.

## Data sharing

Computer code for implementing our model and replicating our results can be found at the author’s GitHub account (https://github.com/AinaRB?tab=repositories). Interactive visualizations of the results are available on the project’s website (https://ainarb.github.io/climate_and_health/). Municipality-wide daily mortality records are publicly available for download at the national Public Health System database (Sistema Único de Saúde, DATASUS) platform, http://www2.datasus.gov.br/DATASUS/index.php?area=0205&id=6937, or from the Epidemiology and Information Department of the Municipal Health Secretariat of São Paulo (CEInfo/SMS-SP),https://www.prefeitura.sp.gov.br/cidade/secretarias/saude/epidemiologia_e_informacao/ Hourly weather measurements can be obtained upon request from the Institute of Astronomy Geophysics and Atmospheric Sciences and University of São Paulo (IAG-USP) platform at http://www.estacao.iag.usp.br/. Air pollution data can be accessed upon request from the CETSB website at https://cetesb.sp.gov.br/ar/qualar/.

## Supplementary Information

Below is the link to the electronic supplementary material.

Supplementary file1 (PDF 3.36 MB)
