# Supplementary material for: Trends in Temperature-associated Mortality in São Paulo (Brazil) between 2000 and 2018: an Example of Disparities in Adaptation to Cold and Heat
Source: J Urban Health. 2022 Nov 10;99(6):1012–26. doi: 10.1007/s11524-022-00695-7 (PMC9727050; doi:10.1007/s11524-022-00695-7)
Supplement: Supplementary file 1 — Supplementary file1 (PDF 3.36 MB) [file 11524_2022_695_MOESM1_ESM.pdf]

Supplementary Material

Trends in temperature-associated mortality in São Paulo (Brazil) between 2000 and 2018: an example of disparities in adaptation to cold and heat

Table of contents

Supplementary Methods........3

SM1. Years of education........3

SM2. Temperature measurements........3

SM3. Sensitivity analyses........3

SM4. Temperature-mortality association indicators........3

Supplementary Figures........5

Fig. S1 Study area and location of the IAG-USP meteorological station........5

Fig. S2 Time series of weather and air pollution variables. 6

Fig. S3 Distribution of the daily mean temperature by year........7

Fig. S4 Time series of death counts........8

Fig. S5 Time series of deaths from all non-external causes by category of vulnerable group. ..9

Fig. S6 Cumulative temperature-mortality association for 2000-2018 by population group ..10

Fig. S7 Graphic representation of the interaction terms by population group. 11

Fig. S8 Annual temperature-mortality association by education group........12

Fig. S9 Association between MMT and annual mean temperature 13

Fig. S10 Association of cRR for extreme heat[a] with annual extreme hot temperatures.........14

Fig. S11 Association of cRR for extreme cold[a] with annual extreme cold temperatures. 15

Fig. S12 Trends of MMT by sensitivity analyses........16

Fig. S13 Trends in the cRR for extreme heat[a] by sensitivity analyses........17

Fig. S14 Trends of the cRR for extreme cold[a] by sensitivity analysis. 18

Supplementary Tables........19

Table S1 Percentage of missing data on years of education by year........19

Table S2 Missing data per population group. 19

Table S4 Sensitivity analyses. 21

Table S5 Sensitivity analyses on cRR 10th and 90th percentile... **Error! Bookmark not defined.**

References........23

Supplementary Methods

SM1. Years of education

Information on years of education was of poor quality (average: 35.6% missing values vs <5% for other covariates, **Table S1**). Hence, it was excluded from the main analyses set. Upon investigation of the proportion of annual missing values, we observed an improvement after 2011 (mean: 24.7%; range: 20.5-28.6%) compared to before 2011 (mean: 43.3%; range: 30.0-50.8%) (**Table S2**). Therefore, we used data post-2011 only to explore the effect modification of years of education on the temperature-mortality association (**Fig. S8**). We aggregated the total years of education into three categories to ensure contrast, i.e., ≤3 years, 3 to 11 years, and ≥11 years. Due to the smaller sample size, it was not possible to investigate educational level by categories of gender, age or ethnic group.

SM2. Temperature measurements

Temperature and relative humidity data were obtained from the Institute of Astronomy Geophysics and Atmospheric Sciences and University of São Paulo (IAG-USP) meteorological station located within the *Parque Estadual dos Fontes do Ipiranga* (coordinates: 23,6512°S, 46,6224°W; elevation: 799.2m, **Figure S1**). Air temperature is recorded hourly by an observer using an Assmann psychrometer and complemented with readings from a Daily Bimetallic Thermograph. Hourly RH is calculated from hourly wet bulb temperature and atmospheric pressure as observed in a barometer, complemented with estimates from a hygrograph with daily rotation.

SM3. Sensitivity analyses

We performed a total of 23 sensitivity analyses. These included changes in: the lag dimension [maximum lag values: 15, 21 and 24, and number of knots: 2 and 3]; the temperature-mortality dimension [the df: 2 and 3, and location of the knots: 75th; 50th and 90th; 25th, 50th and 90th; 25th, 50th, 90th and 99th; 10th, 75th and 90th], the seasonality [number of df per year: 3, 7, 8, 9, 10] and trend adjustment [number of df: 1 and 2], and adjustment for RH [moving average over lag 0, 1 and 2 adjusted as a natural cubic spline with 3 df, and moving average over lag 0 adjusted as a natural cubic spline with 4 df] and PM_{10} [moving average over lag 1, 2 and 3]. See **Table S3** for details. We tested all model parametrizations on all non-external causes of death, including and excluding the linear interaction term. Finally, to evaluate the robustness of our results against alternative model parametrizations, we estimated annual MMT values (**Fig. S12**) and cRR estimates (**Table S4**) resulting from all sensitivity analyses. We found no significant change, supporting the robustness of our model.

SM4. Temperature-mortality association indicators

The mortality risk associated to temperature follows a U-shaped distribution, with the lowest point or the temperature at which a given population shows the lowest risk of death, referred to as minimum mortality temperature (MMT). Generally, the risk of mortality increases as temperatures deviates from the MMT toward the left (cold) or right (heat). The slope of the curve

defines the increment of risk per unit of temperature change, the steeper the slope the larger the increase in risk (**Fig. SM4**). Hence, a decrease in the slope (or flattening of the curve) over time indicates a reduction in the risk increment per unit of temperature change. The temperature-mortality risk curve may, therefore, be used as an indicator of the degree of adaptation to deviances from the MMT. Alternatively, the MMT itself is considered a good indicator for long-term adaptation to the local usual temperatures as it usually shows a good correlation with the local annual mean temperature (AMT) and summer mean temperature [1,2]. Therefore, shifts in the MMT over time have been previously used as an indicator of human long-term adaptation to temperatures[3–5].

These two indicators are essential in developing evidence-based public health interventions such as early warning systems (EWS), just to mention one. EWS are comprehensive systems that combine weather forecast information with epidemiological evidence on the health burden of temperature to define the conditions that need to be met for the activation of action plans to protect the population's health. According to the WHO guidelines[6], the gold standard for EWS is to use epidemiological data to inform the thresholds used to activate such plans. A common approach is to use a static temperature threshold above which, the associated mortality risk is considered to be excessive and preventable. Multiple thresholds may exist to capture different levels of risk, associated to action plans of increasing magnitude and urgency. In order for EWS to be effective, they have to capture the real risk of the population. Thus, understanding whether the temperature-mortality association has changed over time, becomes vital. The selection of the mortality risk used to define the activation thresholds is decided based on the cRR distribution across the temperature continuum and defined in reference to the temperature with the lowest mortality risk recorded or MMT. Thus, variations in the MMT and cRR not only are good indicators of adaptation, but can also flag the need updates and improvements in public health measures such as EWS.

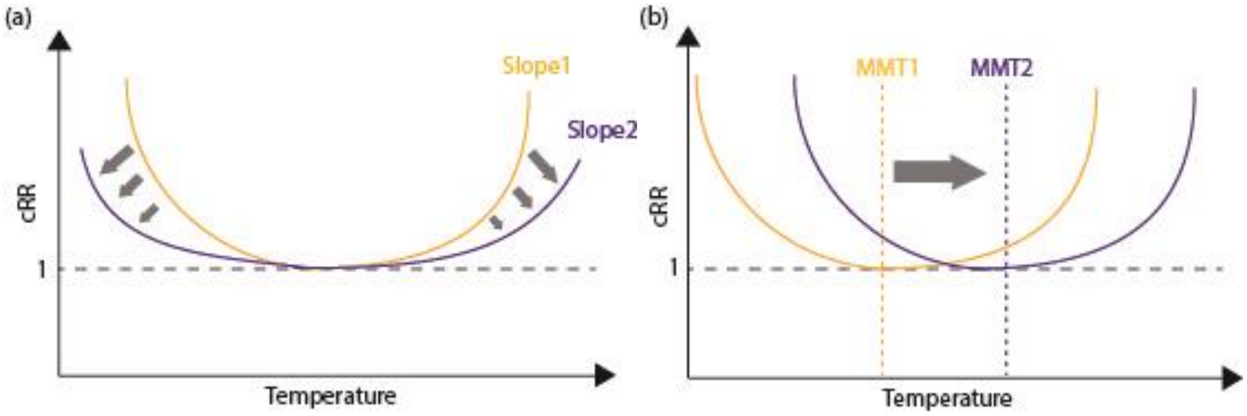

Fig. SM4 Diagram of the two indicators of change used in this study. For two imaginary time points (1, yellow and 2, purple), adaptation can be observed involving (a) a flattening in the cumulative relative risk (cRR) curve suggestive of changes in the mortality impacts of non-optimal temperatures including mild and extreme, for both cold and heat, and (b) a shift of the minimum mortality temperature (MMT), in this case to the right, suggesting that the optimal temperature has increased.

Supplementary Figures

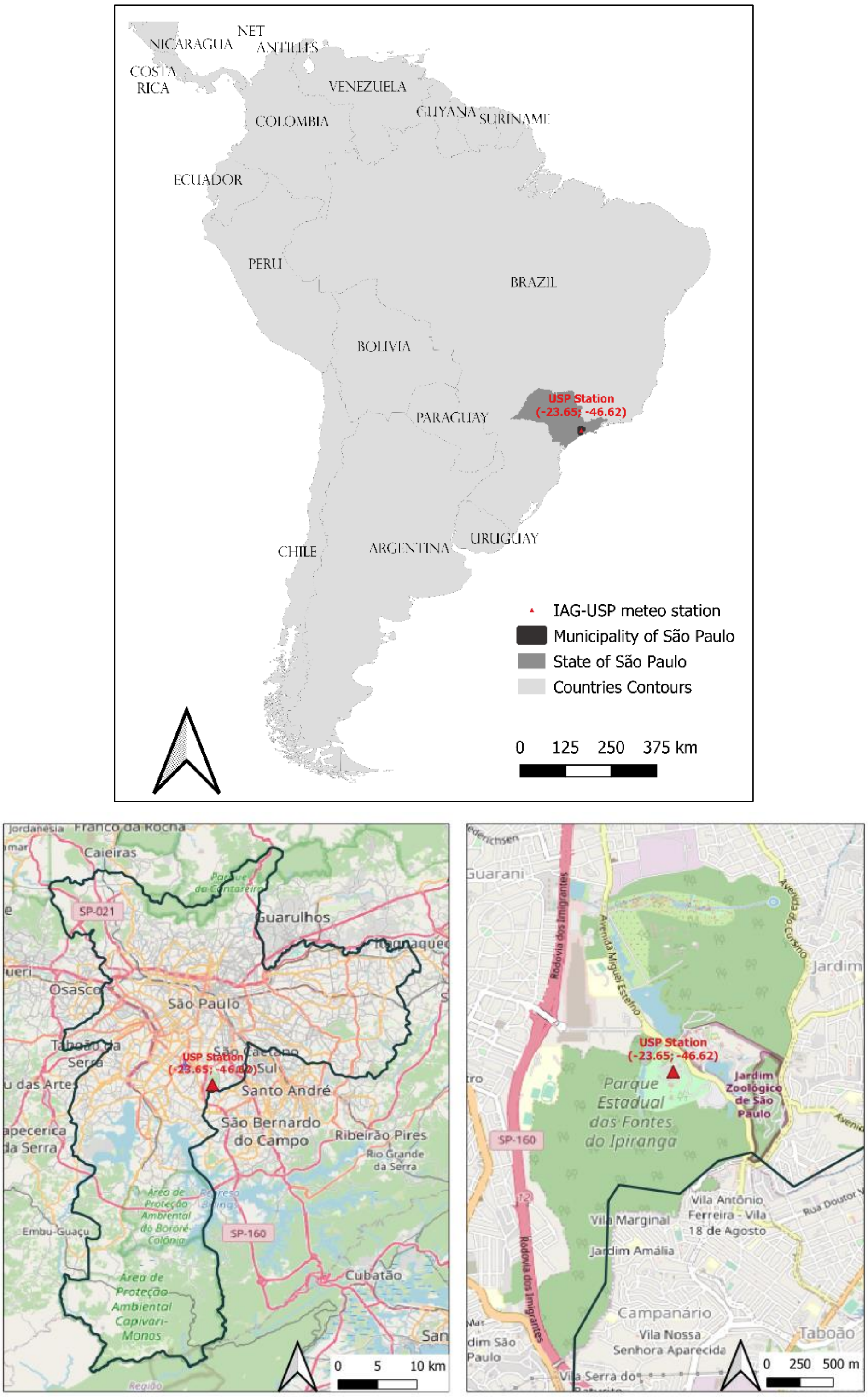

Fig. S1 Study area and location of the IAG-USP meteorological station. (Top) Map of South America with the state (mild grey) and the municipality of São Paulo (dark grey) highlighted. The IAG-USP station is shown in red with the coordinates. (Bottom left) Zoom in of the location of the IAG-USP meteorological station (red triangle) within the municipality of São Paulo (black outline). (Bottom right) Exact location of the station within the *Parque Estadual dos Fontes do Ipiranga.*

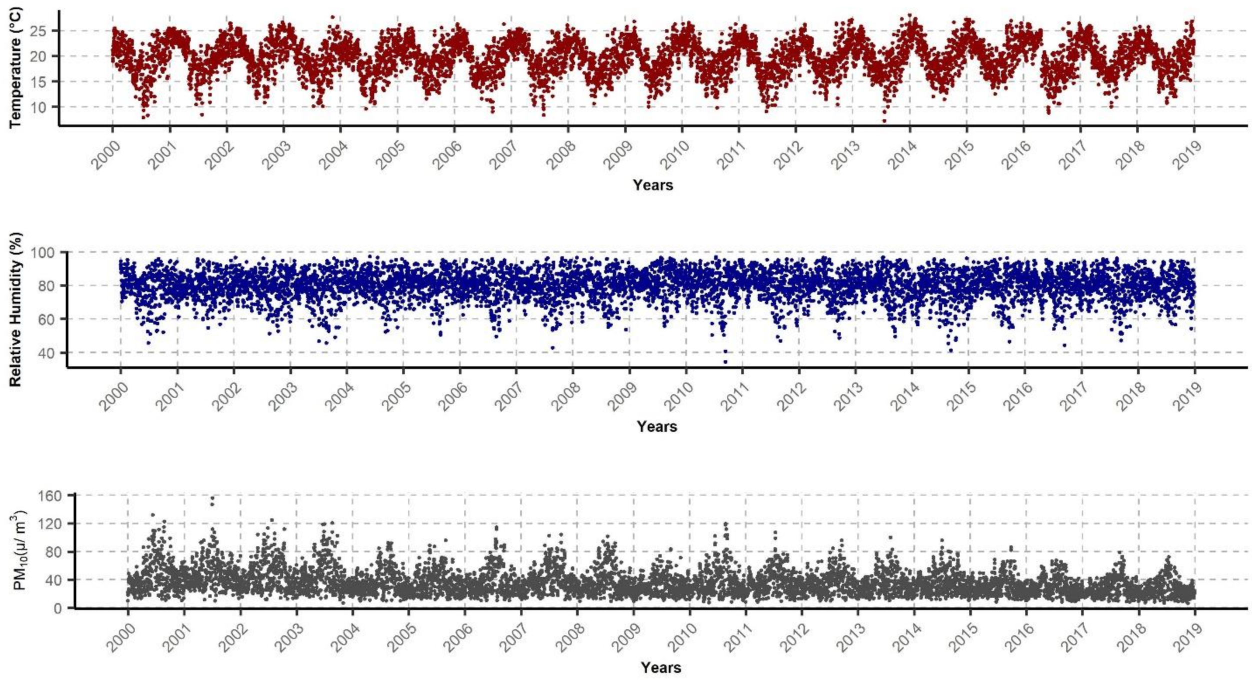

Fig. S2 Time series of weather and air pollution variables. Daily mean temperature (ºC) and relative humidity (%) recorded by the Institute of Astronomy Geophysics and Atmospheric Sciences and University of São Paulo (IAG-USP) meteorological station. PM_{10} daily mean ($\mu g/m^3$) recorded by the regional Environment Agency (CETESB)[7] air pollution automatic network. Municipality of São Paulo, Brazil, 2000 - 2018

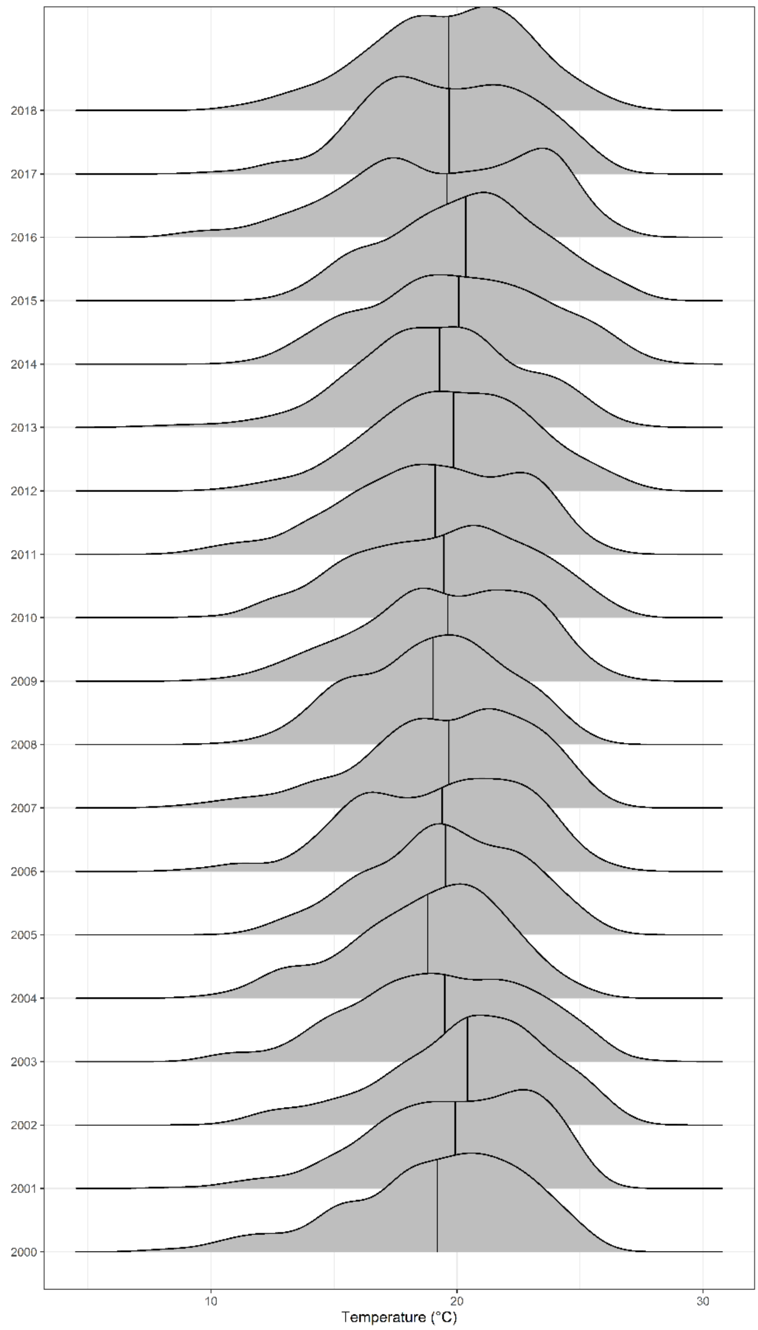

Fig. S3 Distribution of the daily mean temperature by year. The annual average is shown as a solid black vertical line. The average temperature for the entire period is shown as a dashed red vertical line.

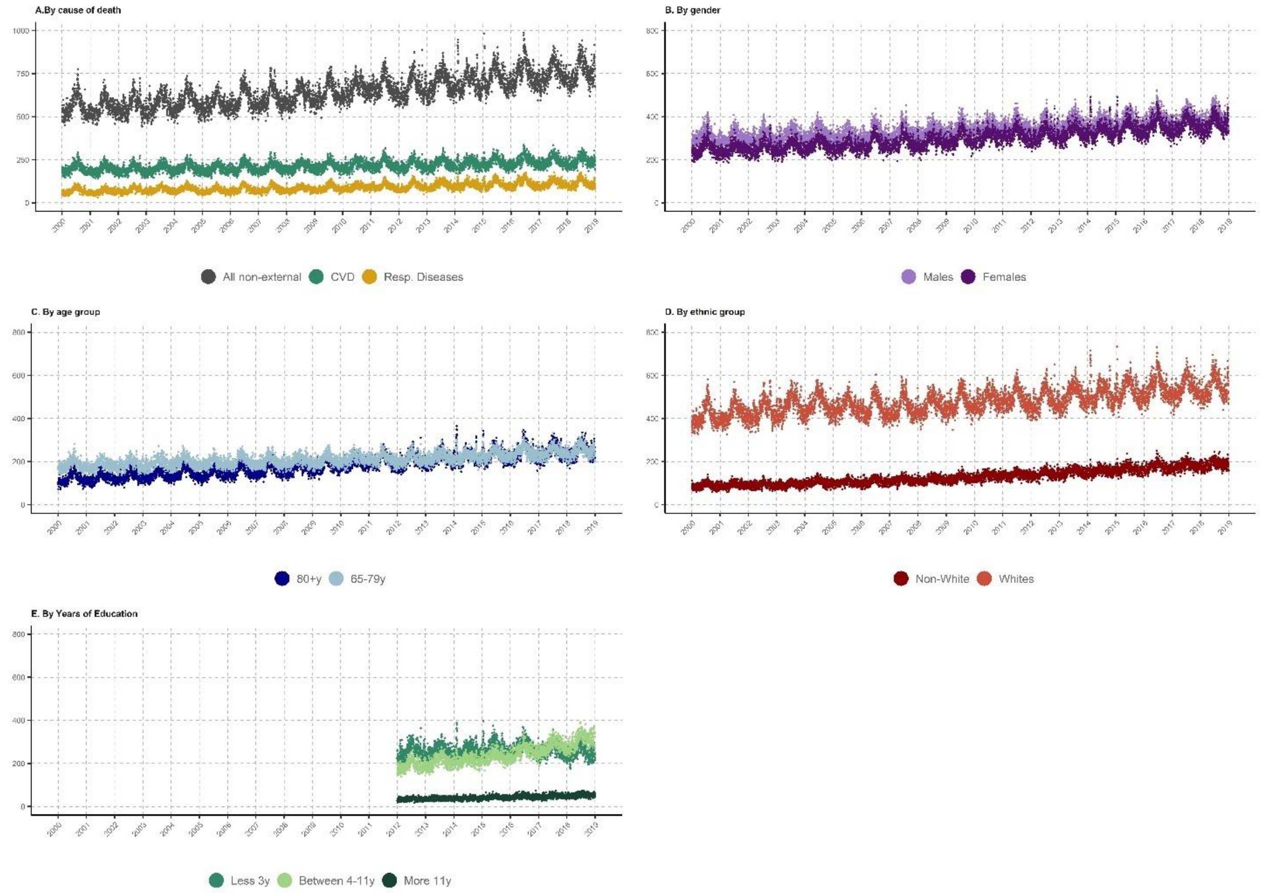

Fig. S4 Time series of death counts. Daily death counts by: (A) cause of death, (B) gender, (C) age group, (D) ethnic group, and (E) years of education (only post-2011, see methods section for justification) recorded in the municipality of São Paulo, Brazil, between 2000 and 2018. Note the *y*-axes are scaled to each population group's counts.

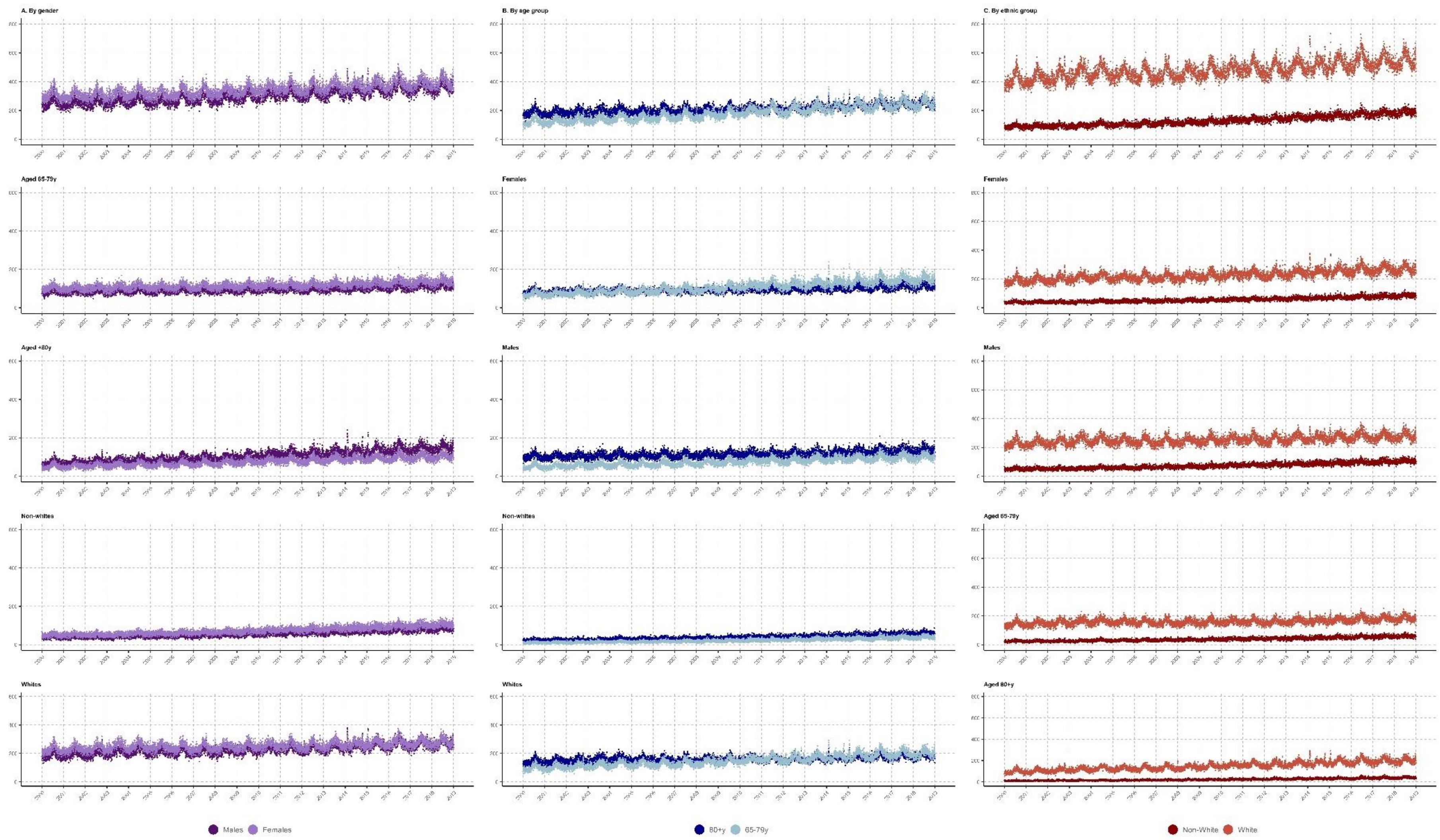

Fig. S5 Time series of deaths from all non-external causes by category of vulnerable group. Daily death counts across groups by: (A) gender, (B) age group, and (C) ethnic group, recorded in the municipality of São Paulo, Brazil, 2000-2018. Note the *y*-axes are scaled to each population group's counts.

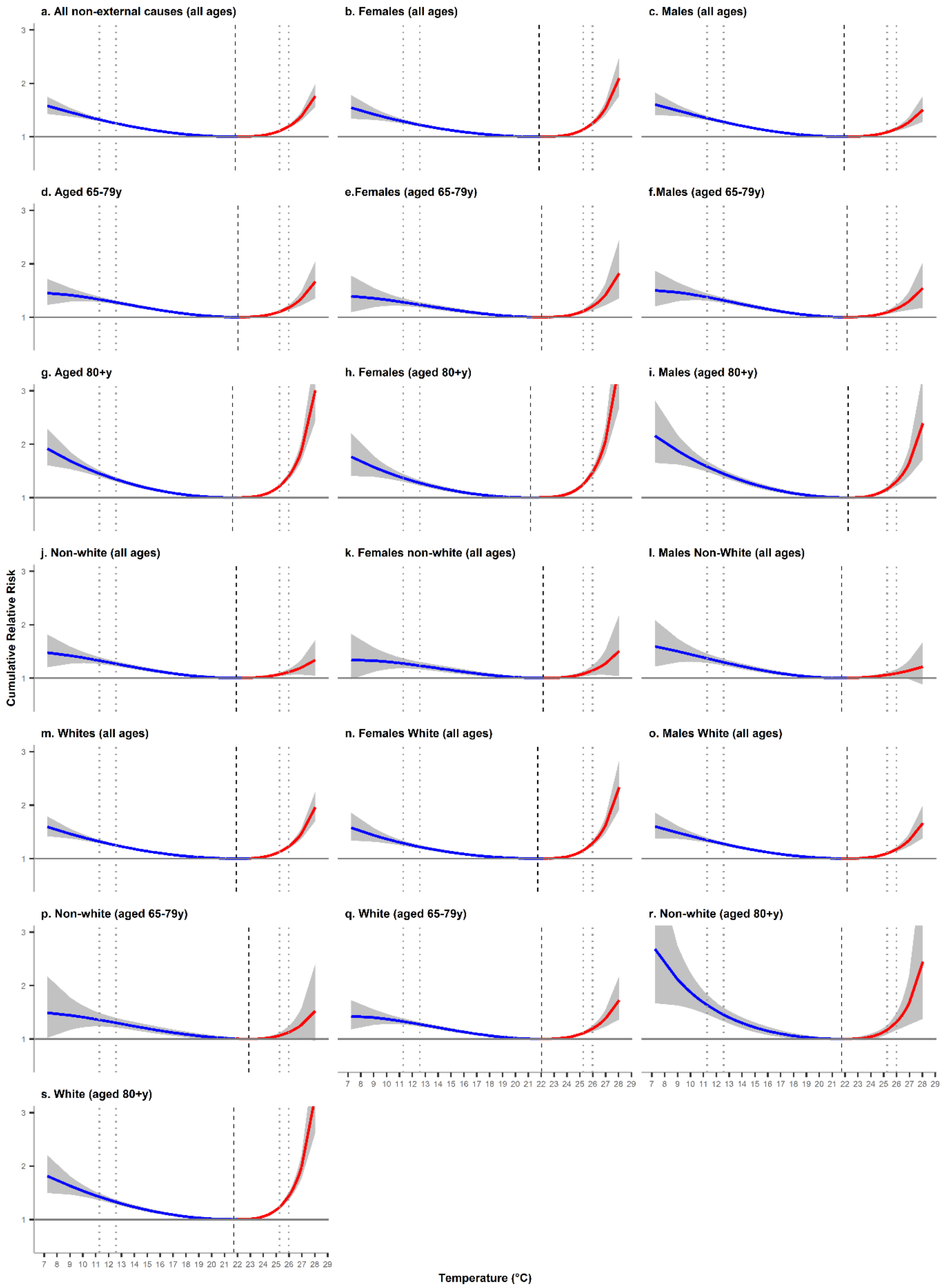

Fig. S6 Cumulative temperature-mortality association for 2000-2018 by population group
Overall cumulative relative risk (cRR, solid line) and 95% CI by age, gender and ethnic group. In blue, the risk to cold temperature and in red to heat. The MMT (dashed), and the 1st, 10th, 90th, and 99th percentiles of the temperature distribution (dotted) are shown as vertical lines. The cRR curves were re-scaled to the group-specific overall MMT.

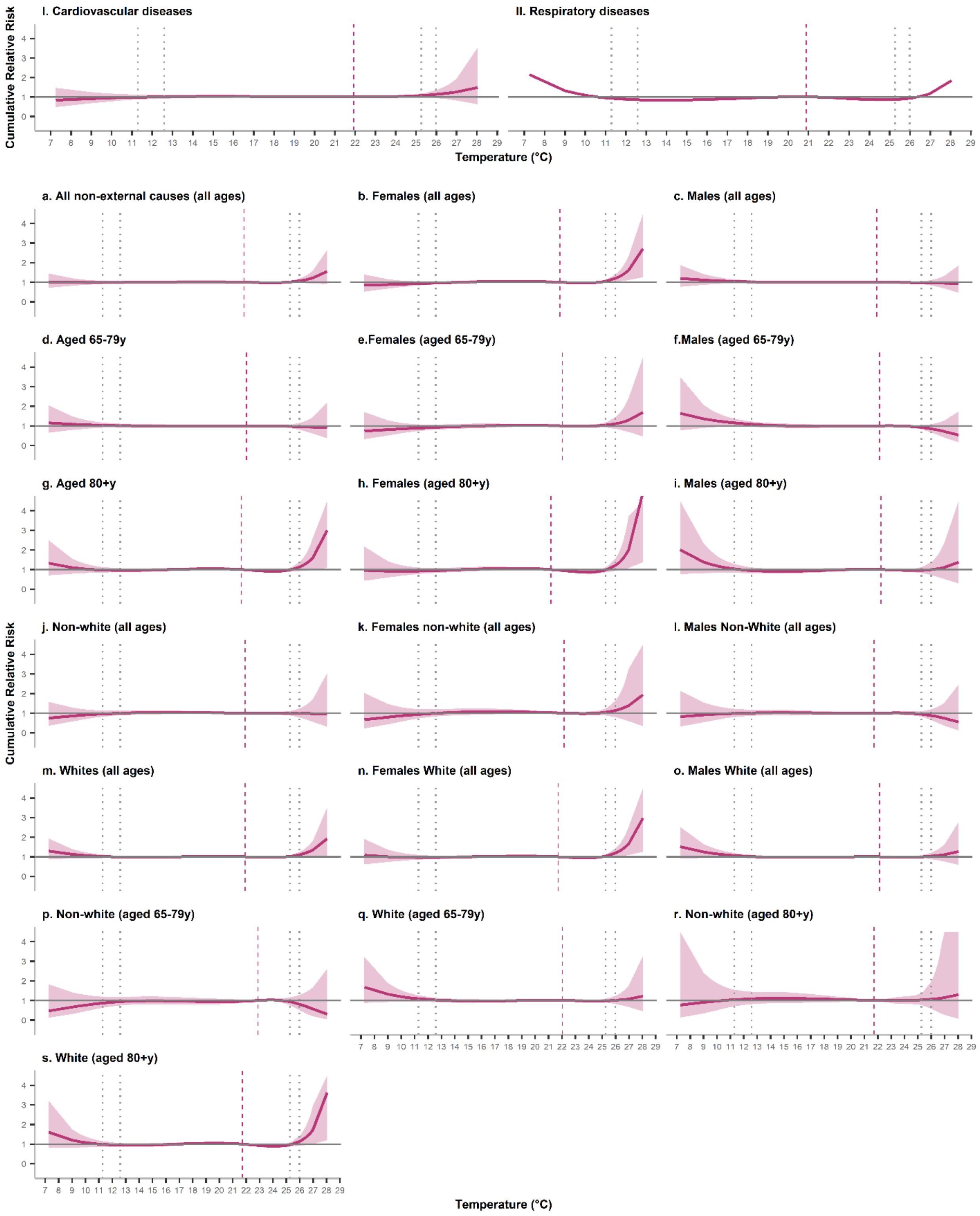

Fig. S7 Graphic representation of the interaction terms by population group. Cumulative relative risk (solid line) and 95% credible intervals (95%CI) by age, gender, and ethnic group. The MMT (dashed), and the 1st, 10th, 90th, and 99th percentiles of the temperature distribution (dotted) are shown as vertical lines. The curves were re-scaled to the MMT from the model without interaction (interpreted as the average across the whole study period).

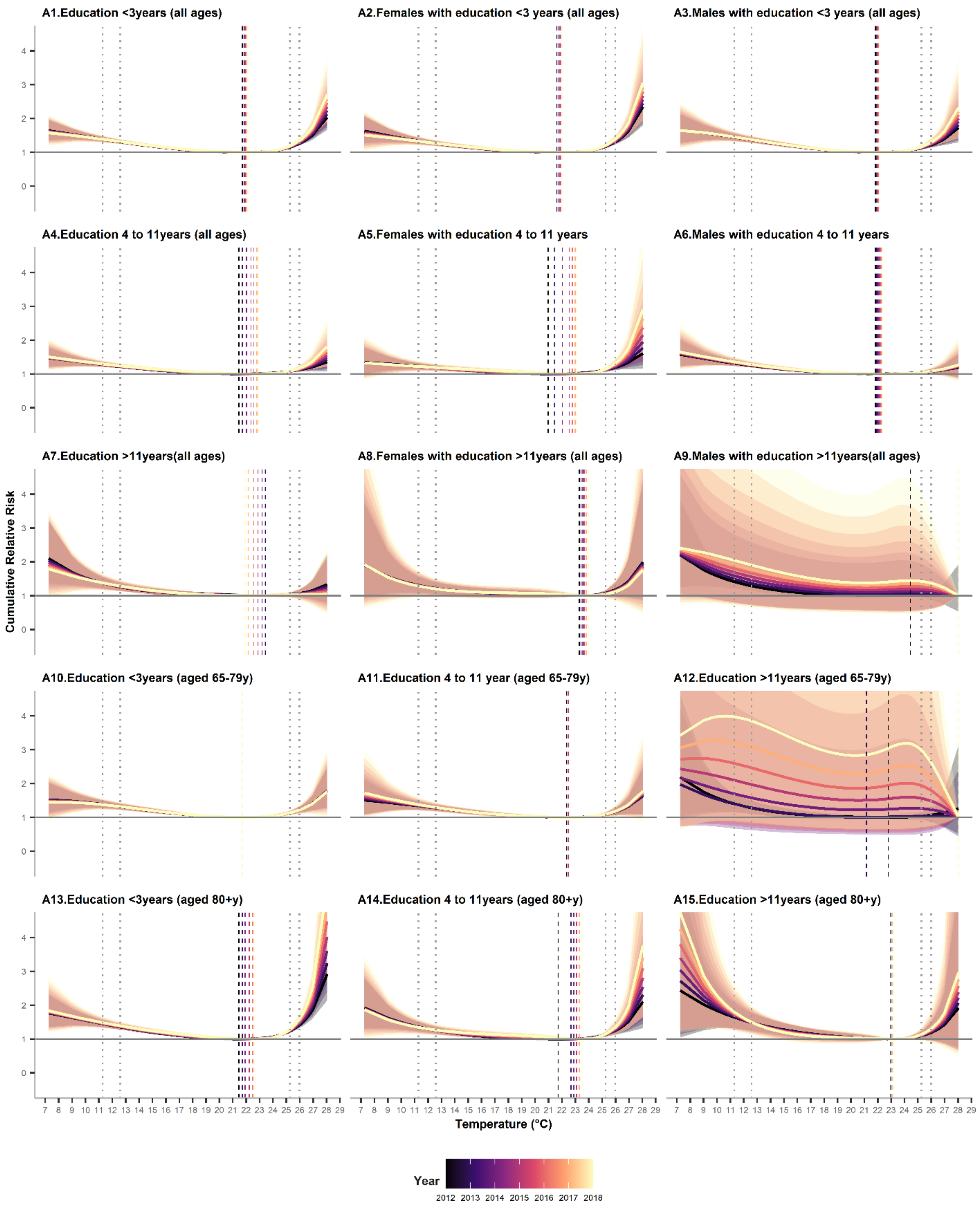

Fig. S8 Annual temperature-mortality association by education group with 95%CI. Annual cumulative relative risk (solid line) and 95%CI (shaded) for the temperature-mortality association by years of education category, gender and age group. The MMT (dashed), and the 1st, 10th, 90th, and 99th percentiles of the temperature distribution (dotted) are shown as vertical lines

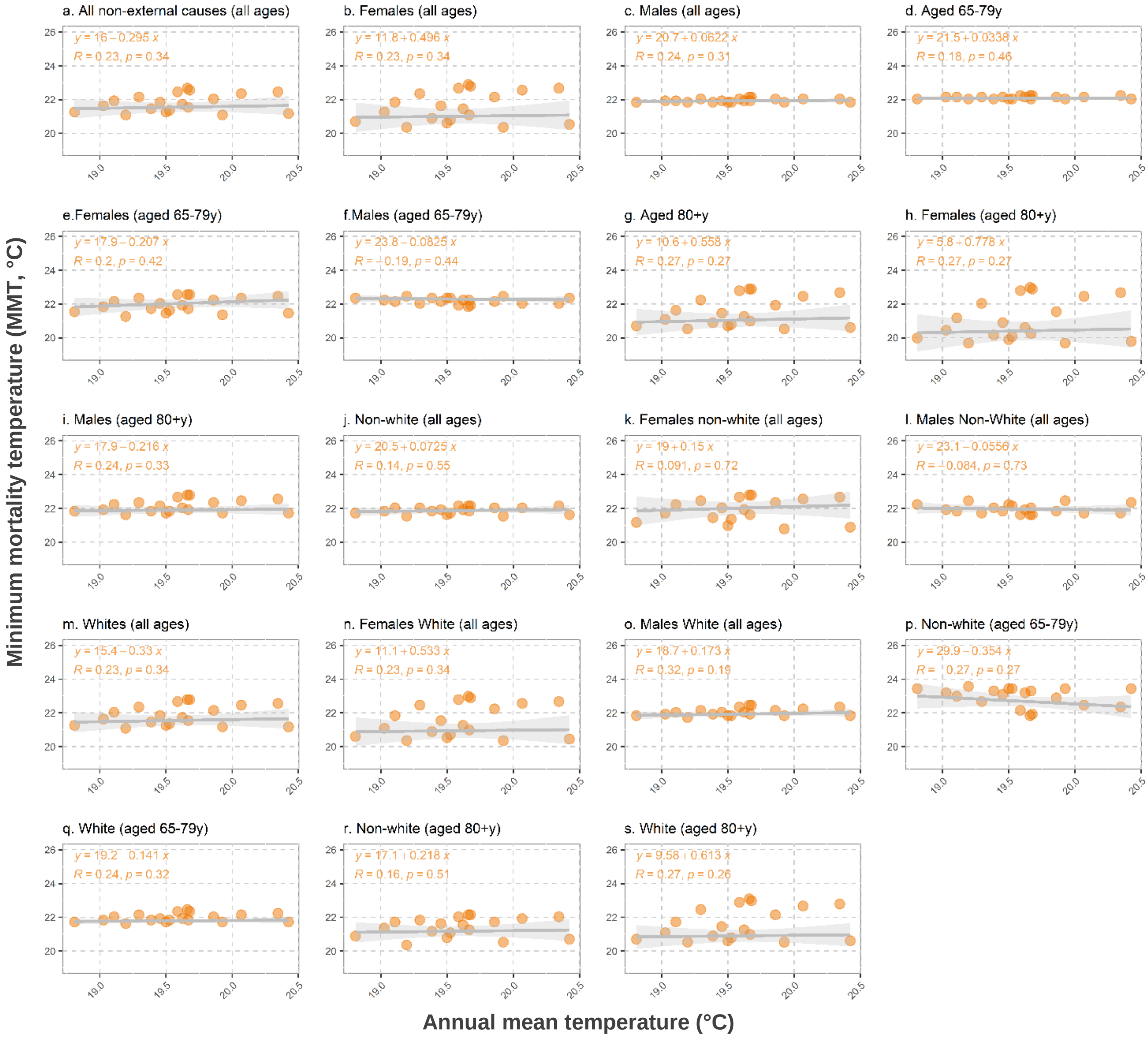

Fig. S9 Association between MMT and annual mean temperature. On the y-axis, the annual MMT. On the x-axis, the annual mean temperature (AMT). Plots or each population group. Plots shown by population group. Regression line and coefficients, Pearson correlation coefficient and p-value shown in orange.

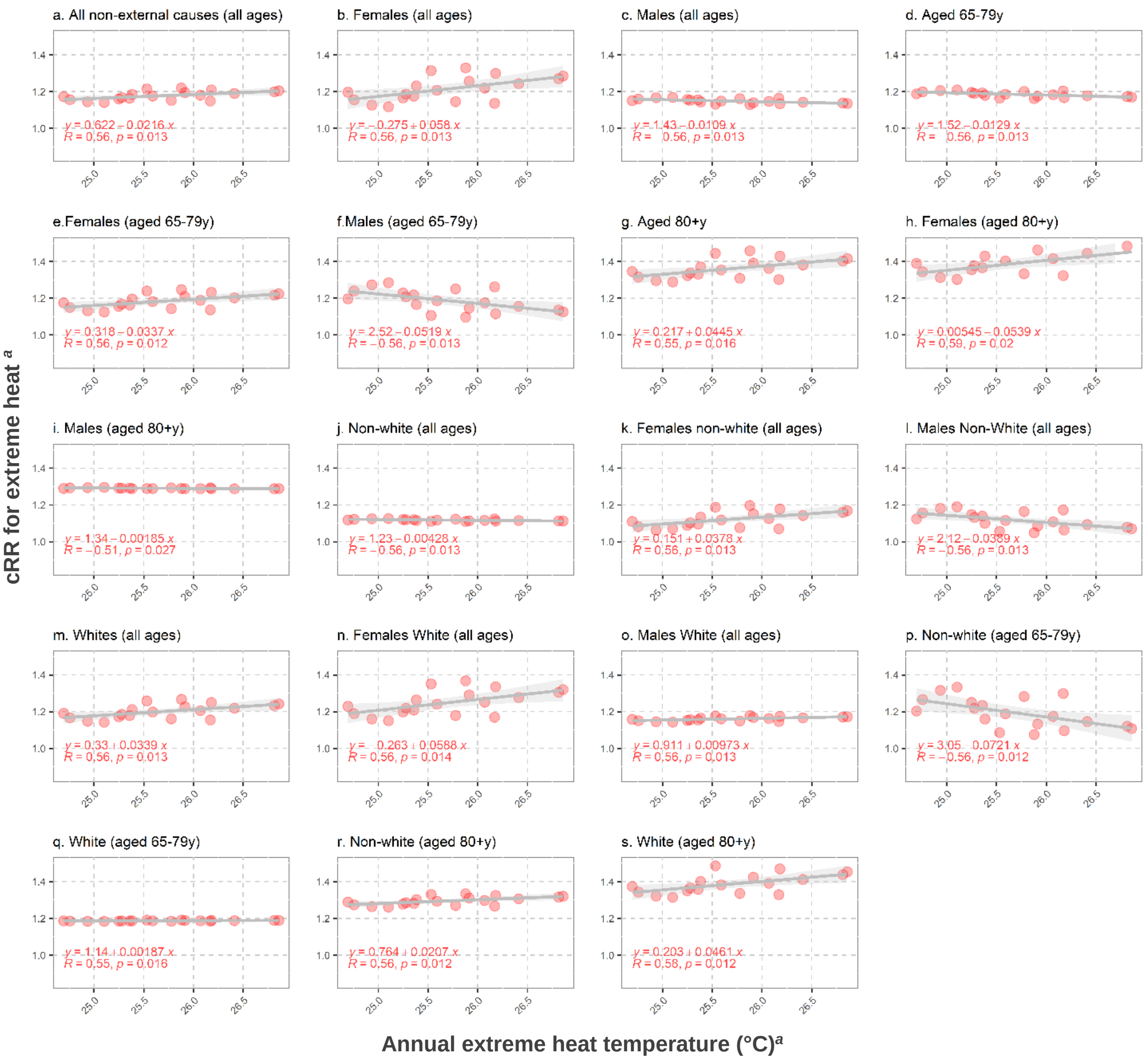

Fig. S10 Association of cRR for extreme heat[a] with annual extreme hot temperatures. On the y-axis, the cRR associated to extreme heat, defined as the cRR associated to the 99th percentile of the annual temperature distribution. On the x-axis, the 99th percentile of annual temperature distribution. Plots shown by population group. Regression line and coefficients, Pearson correlation coefficient and p-value shown in red. [a]Extreme heat: cRR at the 99th percentile vs. MMT.

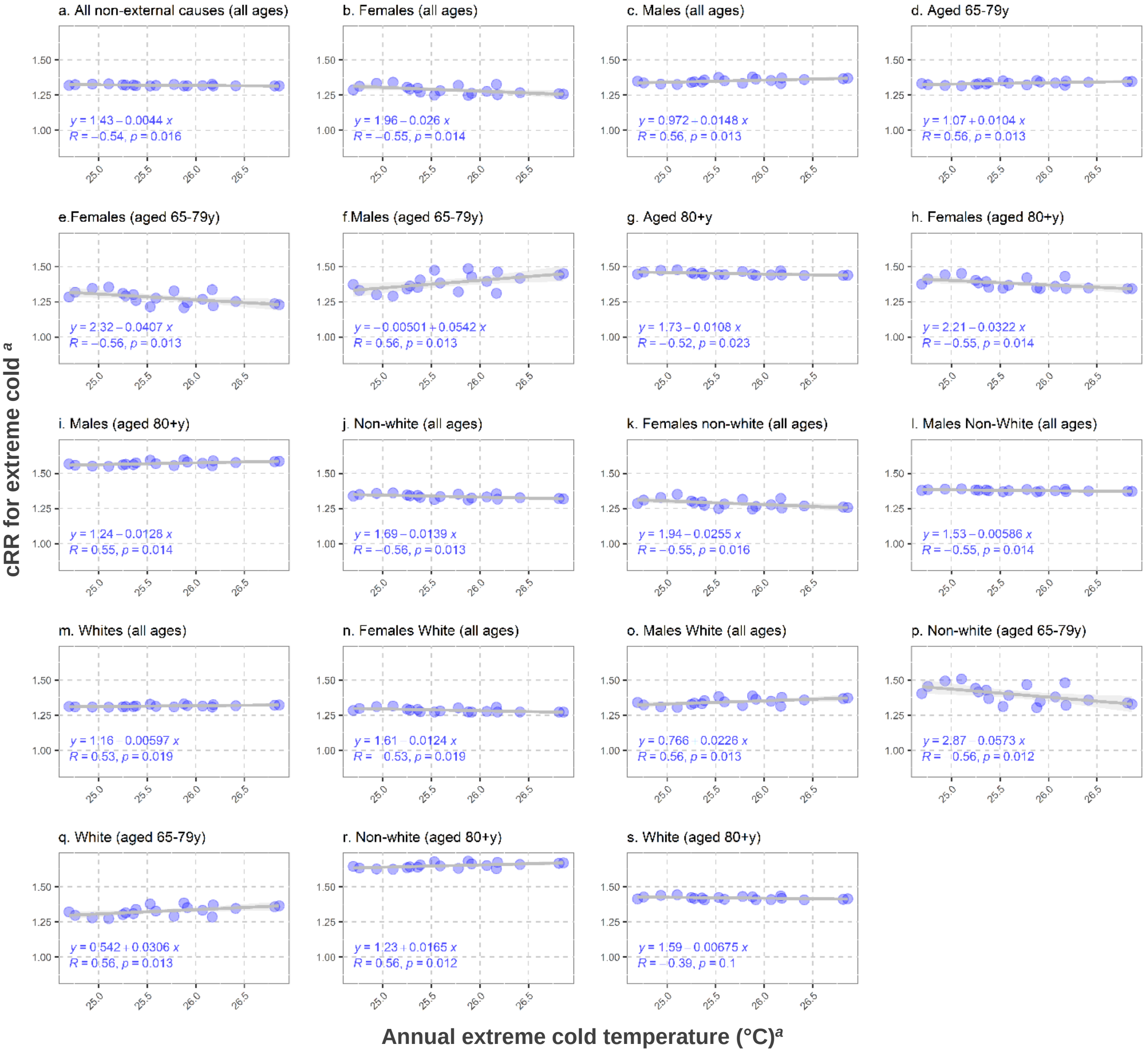

Fig. S11 Association of cRR for extreme cold[a] with annual extreme cold temperatures. On the y-axis, the cRR associated to extreme cold, defined as the cRR associated to the 1st percentile of the annual temperature distribution. On the x-axis, the 1st percentile of the annual temperature distribution. Plots shown by population group. Regression line and coefficients, Pearson correlation coefficient and p-value shown in blue. [a] Extreme cold: cRR at the 1st percentile vs. MMT.

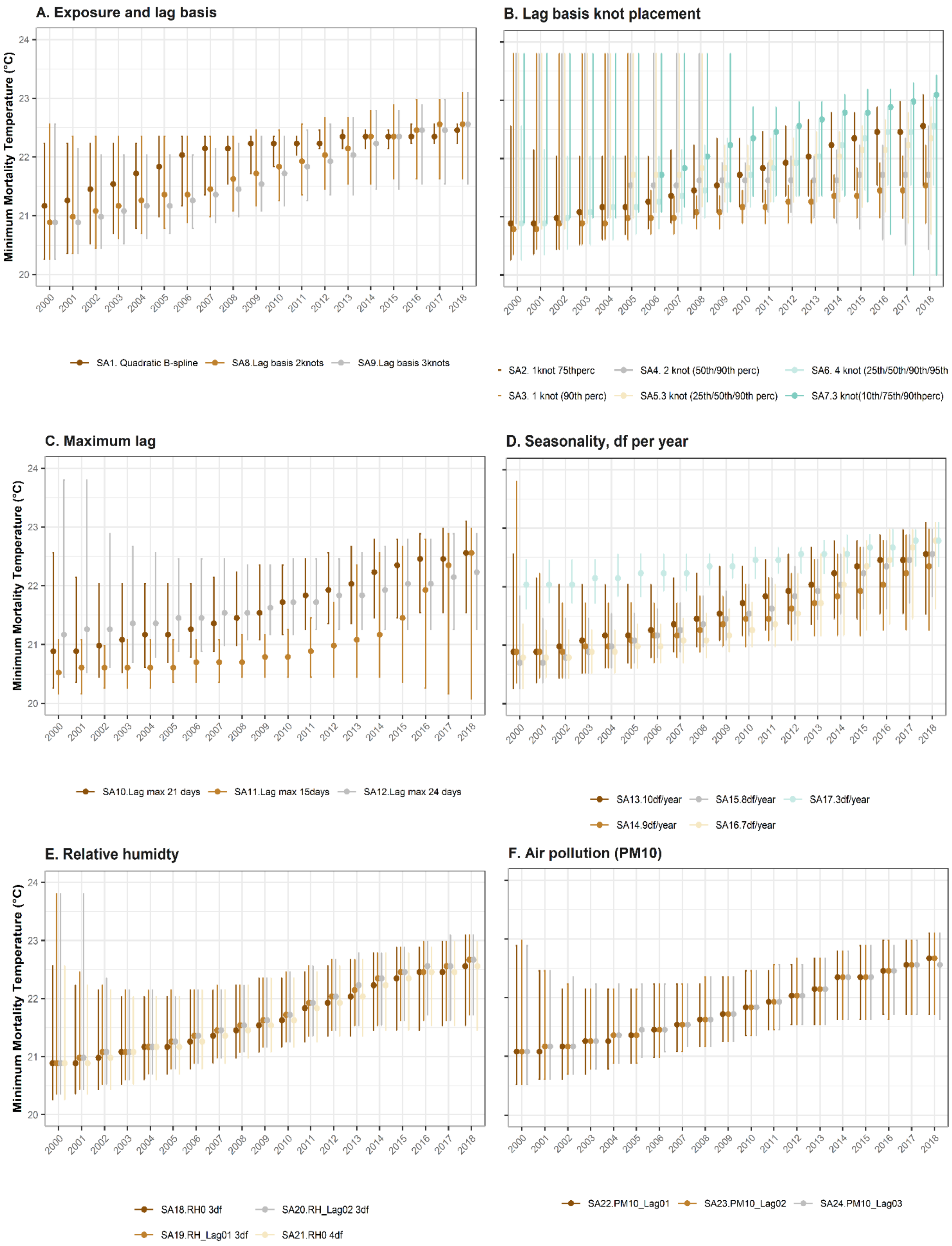

Fig. S12 Trends of MMT by sensitivity analyses. Annual MMT estimates and 95%CI by varying modelling (A to D) choices and controlling for relative humidity (E) and PM_{10} (F). Computed on all-non external causes only. A description of all sensitivity analysis can be found in Table S2.

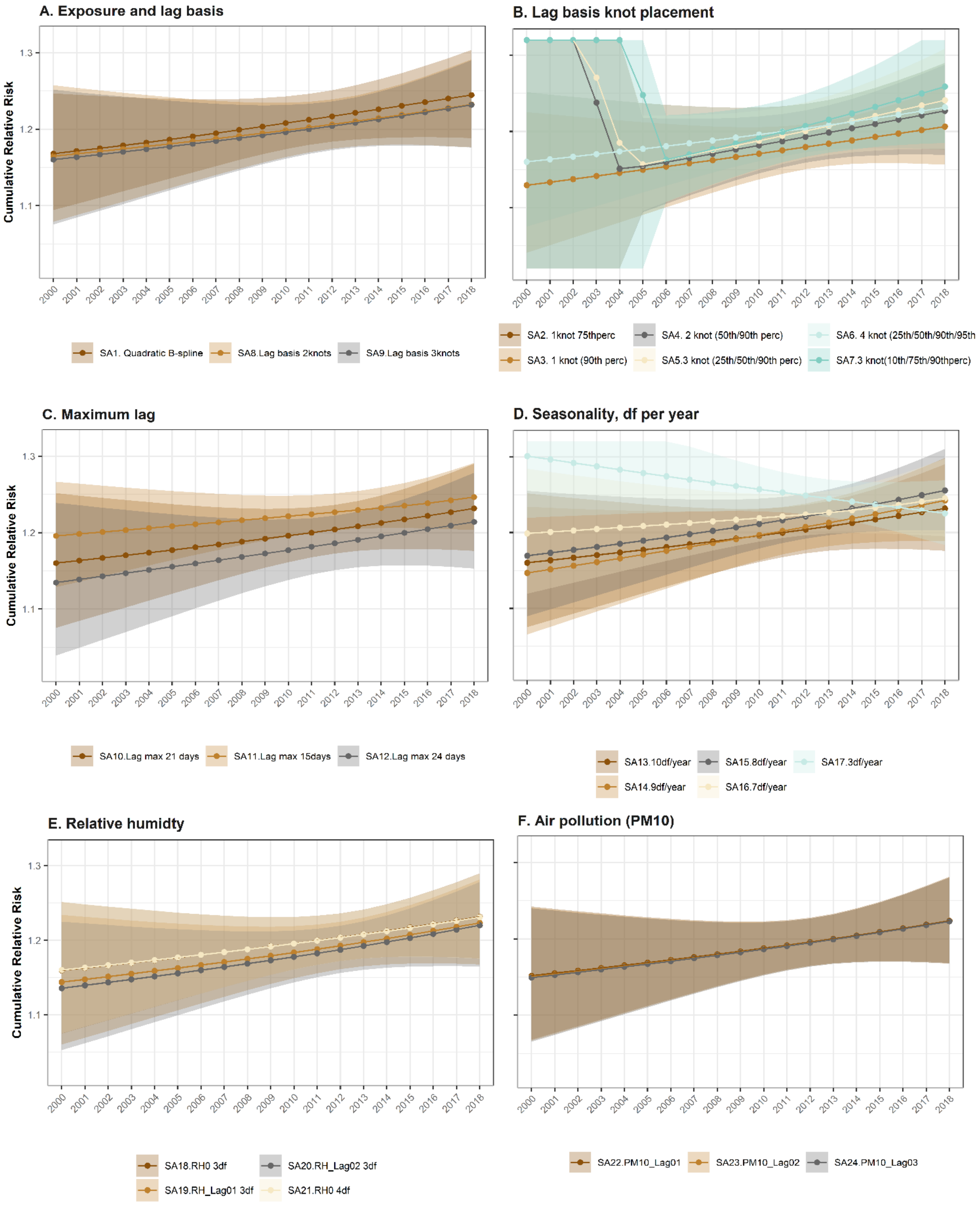

Fig. S13 Trends in the cRR for extreme heat[a] by sensitivity analyses. The cRR and 95%CI are presented by varying modelling (A to D) choices and controlling for relative humidity (E) and PM10 (F). Computed on all-non external causes only. A description all sensitivity analyses can be found in Table S2. [a] Extreme heat: cRR at the 99th percentile vs. MMT.

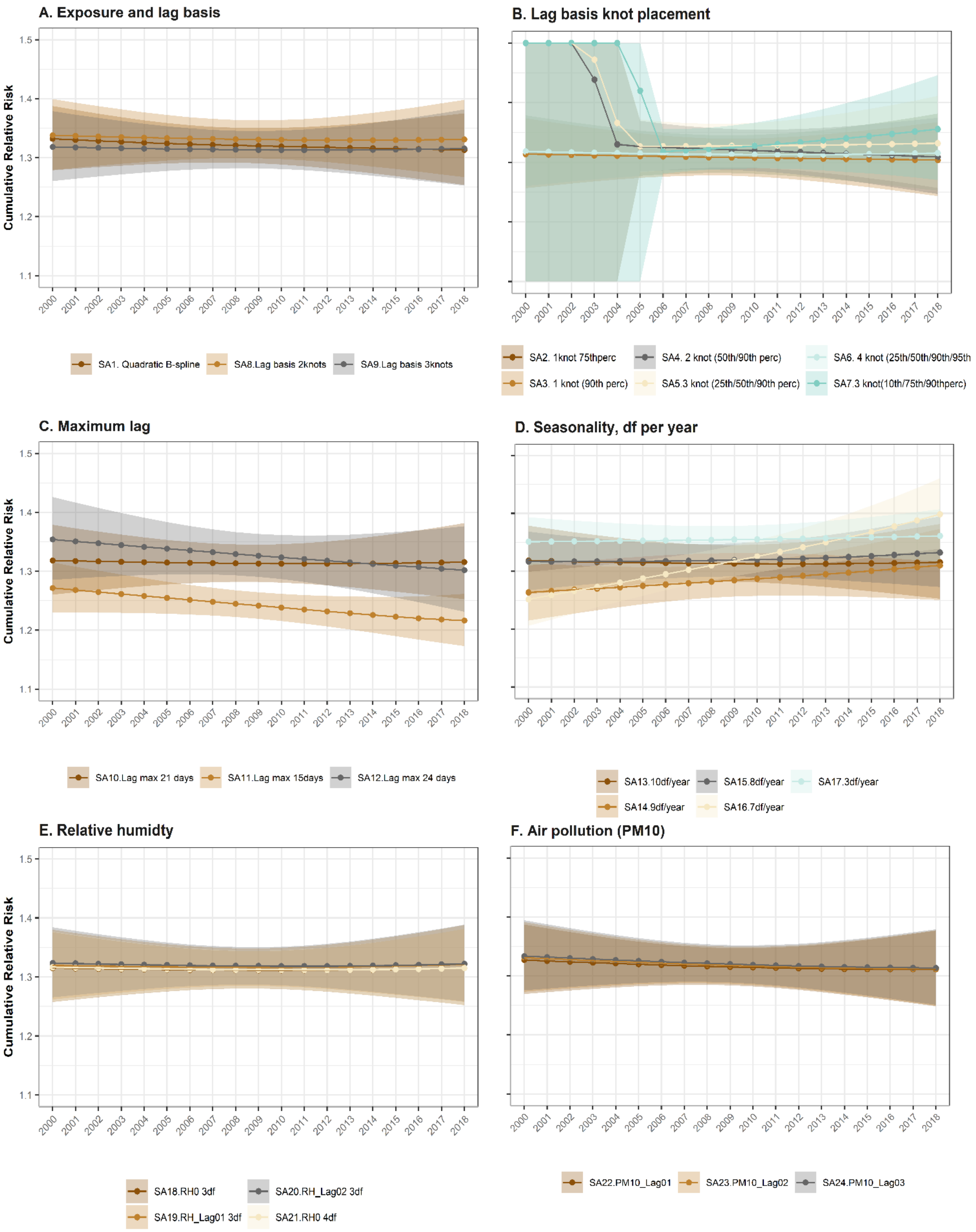

Fig. S14 Trends of the cRR for extreme cold[a] by sensitivity analysis. The cRR and 95%CI are provided by varying modelling (A to D) choices and controlling for relative humidity (E) and PM10 (F). Computed on all-non external causes only. A description all sensitivity analyses can be found in Table S2. [a]Extreme cold: cRR at the 1st percentile vs. MMT.

Supplementary Tables

Table S1 Percentage of missing data on years of education by year, between 2000 and 2018

Year	Missing (n)	Total deaths	Missing Percentage (%)
2000	121,315	238,959	50.8%
2001	117,243	235,987	49.7%
2002	112,544	237,741	47.3%
2003	112,134	240,253	46.7%
2004	111,112	243,984	45.5%
2005	106,879	236,456	45.2%
2006	106,633	243,955	43.7%
2007	102,264	244,653	41.8%
2008	103,382	249,247	41.5%
2009	103,556	256,627	40.4%
2010	97,771	264,951	36.9%
2011	80,802	270,367	29.9%
2012	77,335	270,432	28.6%
2013	77,092	276,980	27.8%
2014	76,542	281,624	27.2%
2015	71,369	287,645	24.8%
2016	67,992	296,359	22.9%
2017	63,867	294,753	21.7%
2018	61,034	298,313	20.5%

Table S2 Missing data per population group. Percentage of records with missing and/or erroneous information, which were consequently excluded from each stratified analysis

Dataset	Missing (%)
Gender	0.0%
Age group	0.3%
Ethnic group	3.7%
Education	35.6%
Gender & Age	0.3%
Gender & Ethnic group	3.7%
Age & Ethnic group	4.0%
Gender & Education	35.6%
Age & Education	35.6%

Table S3 Summary statistics for daily air temperature (°C), air pollution (PM_{10}, µg/m^3), RH (%) and mortality counts and percentage (%) by cause of death, age group, gender, and ethnic group in São Paulo municipality between 2000 and 2018 and by 5-year periods representing the early and late years of the study period.

	All period: 2000-2018		Early: 2000-2004		Late: 2014-2018	
**Environmental data**						
**Temperature (°C)**						
Mean	19.6		19.6		19.9	
Median	19.8		19.9		20.0	
Maximum	28.0		27.6		28.0	
Minimum	7.3		7.9		8.8	
SD	3.3		3.4		3.4	
IQR	4.8		4.7		4.9	
**Air Pollution (PM_{10}, µm)**						
Mean	36.2		43.5		29.8	
Median	32.2		38.9		26.8	
Maximum	156.2		156.2		95.6	
Minimum	6.2		6.7		6.2	
SD	18.1		21.0		13.6	
IQR	21.9		26.3		17.3	
**Relative Humidity (%)**						
Mean	80.0		79.6		79.7	
Median	80.8		80.4		80.6	
Maximum	97.3		97.3		96.3	
Minimum	34.3		45.5		41.2	
SD	8.5		8.5		8.7	
IQR	10.5		10.5		10.7	
**Mortality data (counts)**						
All non-external	4,471,000		1,035,174		1,343,444	
By cause of death ¥						
Cardiovascular	1,492,164	(33.4%)	361,195	(34.9%)	431,911	(32.1%)
Respiratory diseases	614,959	(13.8%)	132,594	(12.8%)	199,971	(14.9%)
By gender						
Females	2,074,790	(46.4%)	466,416	(45.1%)	639,714	(47.6%)
Males	2,395,487	(53.6%)	568,684	(54.9%)	703,471	(52.4%)
By age group ¥						
Aged 65-79yo	1,463,498	(32.7%)	346,241	(33.4%)	435,915	(32.4%)
Aged 80+yo	1,263,237	(28.3%)	240,325	(23.2%)	432,443	(32.2%)
By ethnic group ¥						
Non-Whites	896,669	(20.1%)	170,592	(16.5%)	315,624	(23.5%)
Whites	3,332,295	(74.5%)	800,232	(77.3%)	976,588	(72.7%)
By years of education ¥, *						
Less than 3y	668,220	(14.9%)	-		479,869	(46.4%)
Between 4-11y	614,751	(13.7%)	-		470,037	(45.4%)
More than 11y	109,124	(2.4%)	-		83,213	(8.1%)

RH = relative humidity; SD = Standard deviations; IQR = Interquartile range.

¥ Percentages estimated over the total population (including other groups not shown here and missing values) hence the percentages do not add up to 100%.

*Data shown only for years after 2011 due to high proportion of missing values (>30%) in prior years.

Table S4 Sensitivity analyses. Computed on all-non external causes only by varying modelling choices and controlling for PM_{10} and relative humidity. qAIC values are provided for each sensitivity analysis for both the interaction and the no interaction models. Model of choice in bold

SA	Exposure basis	Exposure basis knots	Lag basis	Lag basis knots	Lag max	DF seasonality	RH term	PM_{10} term	$qAIC_{noint}$	$qAIC_{int}$
1	Quadratic B-spline	1 knot at 75th percentile	cubic B-spline	3 knots equally spaced log scale + intercept	21	ns(time, df=10/year)	-	-	66709.4	66468.7
2	Cubic B-spline	1 knot at 75th percentile	cubic B-spline	3 knots equally spaced log scale + intercept	21	ns(time, df=10/year)	-	-	66684.6	66475.3
3	Cubic B-spline	1 knot at 90th percentile	cubic B-spline	3 knots equally spaced log scale + intercept	21	ns(time, df=10/year)	-	-	66711.5	66475.4
4	Cubic B-spline	2 knot at 50tha and 90th percentile	cubic B-spline	3 knots equally spaced log scale + intercept	21	ns(time, df=10/year)	-	-	66690.2	66495.8
5	Cubic B-spline	3 knot at 25th, 50th, 90th percentile	cubic B-spline	3 knots equally spaced log scale + intercept	21	ns(time, df=10/year)	-	-	66699.8	66578.1
6	Cubic B-spline	4 knot at 25th, 50th, 90th, 95th percentile	cubic B-spline	3 knots equally spaced log scale + intercept	21	ns(time, df=10/year)	-	-	66684.6	66607.9
7	Cubic B-spline	3 knot at 10th, 75th, 90th percentile	cubic B-spline	3 knots equally spaced log scale + intercept	21	ns(time, df=10/year)	-	-	66705.3	66614.9
8	Cubic B-spline	1 knot at 75th percentile	cubic B-spline	2 knots equally spaced log scale + intercept	21	ns(time, df=10/year)	-	-	66823.5	66657.5
9	Cubic B-spline	1 knot at 75th percentile	cubic B-spline	3 knots equally spaced log scale + intercept	21	ns(time, df=10/year)	-	-	66684.6	66657.5
10	Cubic B-spline	1 knot at 75th percentile	cubic B-spline	3 knots equally spaced log scale + intercept	21	ns(time, df=10/year)	-	-	66684.6	66657.5
11	Cubic B-spline	1 knot at 75th percentile	cubic B-spline	3 knots equally spaced log scale + intercept	15	ns(time, df=10/year)	-	-	66882.1	66657.5
12	Cubic B-spline	1 knot at 75th percentile	cubic B-spline	3 knots equally spaced log scale + intercept	24	ns(time, df=10/year)	-	-	66640.5	66657.5
13	Cubic B-spline	1 knot at 75th percentile	cubic B-spline	3 knots equally spaced log scale + intercept	21	ns(time, df=10/year)	-	-	66684.6	66661.1
14	Cubic B-spline	1 knot at 75th percentile	cubic B-spline	3 knots equally spaced log scale + intercept	21	ns(time, df=9/year)	-	-	66843.0	66661.7
15	Cubic B-spline	1 knot at 75th percentile	cubic B-spline	3 knots equally spaced log scale + intercept	21	ns(time, df=8/year)	-	-	66908.7	66669.9
16	Cubic B-spline	1 knot at 75th percentile	cubic B-spline	3 knots equally spaced log scale + intercept	21	ns(time, df=7/year)	-	-	67148.2	66685.1
17	Cubic B-spline	1 knot at 75th percentile	cubic B-spline	3 knots equally spaced log scale + intercept	21	ns(time, df=3/year)	-	-	68386.2	66685.6

qAIC, quasi-Akaike Information Criteria; ns, natural cubic spline; df, degrees of freedom; PM_{10}, particulate matter <10µm.

Table S4 (cont.) Sensitivity analyses. Computed on all-non external causes only by varying modelling choices, and controlling for PM10 and relative humidity. qAIC values are provided for each sensitivity analysis. Model of choice in bold

18	Cubic B-spline	1 knot at 75th percentile	cubic B-spline	3 knots equally spaced log scale + intercept	21	ns(time, df=10/year)	ns(RH0, df=3)	-	66688.3	66689.9
19	Cubic B-spline	1 knot at 75th percentile	cubic B-spline	3 knots equally spaced log scale + intercept	21	ns(time, df=10/year)	ns(RH_lag01, df=3)	-	66640.3	66707.8
20	Cubic B-spline	1 knot at 75th percentile	cubic B-spline	3 knots equally spaced log scale + intercept	21	ns(time, df=10/year)	ns(RH_lag02, df=3)	-	66603.8	66798.0
21	Cubic B-spline	1 knot at 75th percentile	cubic B-spline	3 knots equally spaced log scale + intercept	21	ns(time, df=10/year)	ns(RH0, df=4)	-	66688.9	66820.9
22	Cubic B-spline	1 knot at 75th percentile	cubic B-spline	3 knots equally spaced log scale + intercept	21	ns(time, df=10/year)	-	PM_{10}_lag01	66509.4	66863.1
23	Cubic B-spline	1 knot at 75th percentile	cubic B-spline	3 knots equally spaced log scale + intercept	21	ns(time, df=10/year)	-	PM_{10}_lag02	66509.4	66890.6
24	Cubic B-spline	1 knot at 75th percentile	cubic B-spline	3 knots equally spaced log scale + intercept	21	ns(time, df=10/year)	-	PM_{10}_lag03	66527.7	67119.0
**Main model**	**Cubic B-spline**	**1 knot at 75th percentile**	**cubic B-spline**	**3 knots equally spaced log scale + intercept**	**21**	**ns(time, df=10/year)**	**ns(RH_lag02, df=3)**	**PM_{10}_lag02**	**66501.7**	**68362.2**

qAIC, quasi-Akaike Information Criteria; ns, natural cubic spline; df, degrees of freedom; PM_{10}, particulate matter <10µm.

References

1. Yin, Q., Wang, J., Ren, Z., Li, J. & Guo, Y. Mapping the increased minimum mortality temperatures in the context of global climate change. *Nat. Commun.* **10**, 1–8 (2019).
2. Tobías, A. *et al.* Geographical Variations of the Minimum Mortality Temperature at a Global Scale. *Environ. Epidemiol.* **5**, e169 (2021).
3. Barrett, J. R. News | Science Selections Increased Minimum Mortality Temperature in France. *Sci. Sel.* **123**, 184 (2015).
4. Åström, D. O., Tornevi, A., Ebi, K. L., Rocklöv, J. & Forsberg, B. Evolution of Minimum Mortality Temperature in Stockholm, Sweden, 1901–2009. *Environ. Health Perspect.* **124**, 740–744 (2016).
5. Weitensfelder, L. & Moshammer, H. Evidence of Adaptation to Increasing Temperatures. 1971–1975 (2020).
6. World Health Organization(WHO) & World Meteorological Organization (WMO). *Heatwaves and Health: Guidance on Warning-System Development*. (2015).
7. QualAR. QualAR - Qualidade do AR. *a Rede De Medição* 1 https://cetesb.sp.gov.br/ar/qualar/ (2019).
